# A resonance Rayleigh scattering sensor for sensitive differentiation of telomere DNA length and monitoring special motifs (G-quadruplex and i-motif) based on the Ag nanoclusters and NAND logic gate responding to chemical input signals

**DOI:** 10.1186/s12951-018-0407-5

**Published:** 2018-10-09

**Authors:** Shuai Wang, Fei Qu, Wenli Han, Jinmao You

**Affiliations:** 1 The Key Laboratory of Life-Organic Analysis, Qufu Normal University, Qufu, 273165 Shandong China; 2 The Key Laboratory for Colloid and Interface Chemistry of Education Ministry, Shandong University, Jinan, 250100 Shandong China; 3 Laboratory Animal Center, Chongqing Medical University, Chongqing, China; 4 Northwest Institute of Plateau Biology, Chinese Academy of Sciences, Xining, 810001 China

**Keywords:** Ag nanoclusters, Telomere DNA length, G-quadruplex, i-motif, DNA logic gate

## Abstract

**Background:**

Differentiation of telomere length is of vital importance because telomere length is closely related with several deadly diseases such as cancer. Additionally, G-quadruplex and i-motif formation in telomeric DNA have been shown to act as a negative regulator of telomere elongation by telomerase in vivo and are considered as an attractive drug target for cancer chemotherapy.

**Results:**

In this assay, Ag nanoclusters templated by hyperbranched polyethyleneimine (PEI–Ag NCs) are designed as a new novel resonance Rayleigh scattering (RRS) probe for sensitive differentiation of telomere length and monitoring special motifs (G-quadruplex and i-motif). In this assay, free PEI–Ag NC probe or DNA sequence alone emits low intensities of RRS, while the formation of PEI–Ag NCs/DNA complexes yields greatly enhanced RRS signals; however, when PEI–Ag NCs react with G-quadruplex or i-motif, the intensities of RRS exhibit slight changes. At the same concentration, the enhancement of RRS signal is directly proportional to the length of telomere, and the sensitivity of 64 bases is the highest with the linear range of 0.3–50 nM (limit of detection 0.12 nM). On the other hand, due to the conversion of telomere DNA molecules among multiple surrounding conditions, a DNA logic gate is developed on the basis of two chemical input signals (K^+^ and H^+^) and a change in RRS intensity as the output signal.

**Conclusion:**

Our results indicate that PEI–Ag NCs can serve as a novel RRS probe to identify DNA length and monitor G-quadruplex/i-motif through the different increasing degrees of RRS intensity. Meanwhile, the novel attributes of the nanoprobe stand superior to those involving dyes or labeled DNA because of no chemical modification, low cost, green, and high efficiency.

**Electronic supplementary material:**

The online version of this article (10.1186/s12951-018-0407-5) contains supplementary material, which is available to authorized users.

## Background

Telomeres, which are supramolecular structures at the ends of eukaryotic chromosomes, play a vital role in protecting the cell from recombination and degradation [1, 2]. Human telomere DNA is typically 5–8 kilobases (kb) in length with a single-strand 3′-overhang of 100–200 bases [3]. The telomere length may be used in the prognosis of malignancy. For example, in normal somatic cells, telomeres shorten progressively after each round of cell division until they reach a critical size, producing cell senescence and apoptosis [4]. But in cancer cells, there is a wide range of variability for the telomere length, which depends on the balance between the telomere shortening from cell division and telomere elongation because of the telomerase activity [5]. Thus, the evaluation of telomere length is important in understanding clinical significance of the telomere. To date, there are plenty of methods to measure telomere length, including polymerase chain reaction [6], hybridization protection assay [7], situ hybridization [8], flow cytometry [9], primed in situ [10] and single telomere length analysis [11]. Nevertheless, these methods require a large amount of starting material (0.5–5 μg DNA), and specialized, expensive equipment. Additionally, because of the high viscosity of longer telomere DNA, the target structures of the most assays are the single quadruplex units formed by short telomeric sequences (typically 21–26 nt). Hence, very few data are available on the binding properties of longer DNA telomeric sequences.

On the other hand, human telomere DNA consists of tandem repeats of the sequence d (T_2_AG_3_)_n_ (G-rich strand) and d (C_3_TA_2_)_n_ (C-rich strand). The G-rich strand can switch into a quadruplex conformation (G-quadruplex) by Hoogsteen hydrogen bonding [12, 13] and the complementary C-rich strand may form the so-called i-motif with intercalated C–C^+^ base pairs [14, 15]. Recently, it has been reported intramolecular G-quadruplex and i-motif structures from human telomeric DNA [16, 17]. The structures and the stability of the G-quadruplex depend on the metal cations, such as Na^+^, K^+^ [18, 19]. While the stability of i-motif is sensitive to pH, C-rich telomeric repeats can form a stable i-motif structure at acidic pH [20]. Utilizing the polymorphism of telomere DNA molecules produced by environmental factors, Sugimoto et al. modified Rhodamine Green to C-rich strand as the fluorescent probe and 4-(4-dimethylaminophenylazo) benzoic acid to G-rich strand as the fluorescent quencher, developing a DNA logic gate [21].

Additionally, G-quadruplex and i-motif formation in telomeric DNA have been shown to act as a negative regulator of telomere elongation by telomerase in vivo and are considered as an attractive drug target for cancer chemotherapy [15, 22]. It is important to engineer structure- specific G-quadruplex inducing/distinguishing agents for targeted therapeutic and diagnostic applications. Up to now, many organic dyes and small molecules, including malachite green [23], crystal violet [24], thioflavin T [25] and protoberberine [26] have been demonstrated to be useful in sensing quadruplex motif through fluorescence signal. However, for i-motif, there are a limited number of materials that display a strong modulation in fluorescence behavior.

In recent years, resonance Rayleigh scattering (RRS), as an analytical technique, has been obtained much attention because of the sensitivity, rapidity and simplicity. RRS is an absorption-rescattering process produced by the resonance between the Rayleigh scattering and the light absorption with identical frequency [27]. The molecular size, shape, conformation and interfacial properties can influence the scattering intensity [28]. Thus, RRS can provide available information concerning the study of the interaction of biological macromolecules and the molecular recognition. For example, Li’s group discriminated a parallel-stranded G-quadruplex from DNA with other topologies and structures by the RRS method [29]. In addition, the RRS technique has been widely applied to the determination of surfactants [30], metal ions [31], proteins [32], etc.

Herein, we develop a highly sensitive and rapid sensing strategy using Ag nanoclusters templated by polyethyleneimine (PEI), abbreviated as PEI–Ag NCs, as a RRS probe for the identification of telomere length (5′-AG_3_(T_2_AG_3_)_n_-3′, n=1, 3, 6, 10, G-rich strand; the complementary sequence, 5′-C_3_T(A_2_C_3_T)_n_-3′, n=1, 3, 6, 10, C-rich strand) and monitoring G-quadruplex and i-motif. It is found that RRS intensity of free PEI–Ag NCs or telomere DNA is very weak; however, when PEI–Ag NCs interact with telomere DNA, the RRS intensity of the system increases remarkably. Typically, the enhancement of RRS signal is directly proportional to the length of telomere, and telomere DNA containing 64 bases shows the best sensitivity with the linear range of 0.3–50 nM. However, when PEI–Ag NCs react with G-quadruplex or i-motif, the intensities of RRS exhibit slight changes. Thus, a new concept is established for a DNA logic gate through two chemical input signals (K^+^ and H^+^) and the changes in RRS intensity as the output signal. The novel attributes of the nanoprobe on distinguishing special DNA-motif here stand superior to those involving dyes or labelled DNA because of no chemical modification, low cost, green and high efficiency.

## Methods

### Chemicals and reagents

DNA oligonucleotides used in this work (Additional file 1: Table S1) were synthesized by Sangon Biotechnology Co., Ltd. (Shanghai, China). They were purified using the ULTRAPAGE method. The concentrations were measured at 260 nm in ultraviolet–visible (UV–vis) spectrophotometer using extinction coefficients supplied by the manufacturer. Silver nitrate (AgNO_3_), hyperbranched polyethyleneimine (PEI, Mw=600), formaldehyde (HCHO), acetic acid (HAC), trihydroxymethyl aminomethane (Tris), sodium acetate (NaAC), potassium acetate (KAC) were purchased from Aladdin (Shanghai, China). All chemicals were used as received without further purification. Ultrapure water (18.25 MΩ cm) was used throughout all the experiments.

### Instruments

The RRS intensities and spectra were performed on a Hitachi F-7000 fluorescence spectrophotometer (Japan) with a 1 cm × 1 cm quartz cuvette and the slit (EX/EM) was 10.0 nm/10.0 nm; the PMT voltage was 400 V. The UV–vis absorption spectra were obtained on a Cary 300 Bio UV–visible spectrophotometer. The pH values of solutions were measured using a pH meter (Mettler Toledo FE 20, Switzerland). The circular dichroism (CD) spectra were measured from 320 to 220 nm on a Jasco J-810 spectropolarimeter (Japan). Zeta (ζ) potential was measured on a ZetaSizer Nano ZS90 (Malvern Instrument, Worcs, UK).

### Preparation of PEI–Ag NCs

In a typical procedure, PEI was first dissolved in deionized water by stirring for 2 min; then 150 μL AgNO_3_ (0.1 M) was added and the solution was stirred for 2 min. Subsequently, 93 μL HCHO solution (1 M) was added under vigorous stirring and the color of the mixture changed from colorless to yellow, indicating the formation of PEI-capped Ag nanoclusters. It should be noted that the synthesis of PEI–Ag NCs was accorded to our previous report [33], and the optimal ratio of PEI:Ag^+^ was 0.8:1; in this case the PEI was fully bond with Ag^+^. Therefore, the obtained PEI–Ag NCs were not further purified in this work.

### DNA pretreatment

These oligonucleotides were first dissolved in the buffer solution (10 mM Tris-HAC solution of pH 7.4). Next, the solution was heated to 95 °C for 5 min (to dissociate any intermolecular interaction) and then rapidly cooled in ice for 20 min.

Formation of intramolecular G-quadruplexes: The prepared DNA solution was dissolved in 10 mM Tris-HAC buffer (pH 7.4), containing 50 mM KAC. Subsequently, the mixture incubated at 4 °C for 12 h.

Formation of i-motif: In brief, we dissolved the sample in 10 mM NaAC-HAC buffer (pH 5.0). And then, the solution incubated at 4 °C for 12 h.

### RRS Measurement of telomere DNA

In a typical DNA assay, 0.1 μL mL^−1^ PEI–Ag NCs, 130 μL Tris-HAC buffer solutions (10 mM, pH 7.4) and a calculated amount of telomere DNA were mixed together with vigorous stirring. After 2 h at 4 °C, the RRS spectra of the mixture were recorded with synchronous scanning at λex = λem = 220 nm. The RRS intensities of PEI–Ag NCs solutions in the absence (I_0_) and the presence (I) of telomere DNA were recorded, and the ∆*I*_RRS_ = I − I_0_ was calculated.

## Results and discussion

### Synthesis and characterization of PEI–Ag NCs

The Ag NCs templated by PEI (MW=600) were synthesized and characterized carefully in our previous report [33]. As shown in Additional file 1: Figure S1, the PEI–Ag NCs exhibit the maximum emission at 455 nm when excited at 375 nm; two absorption peaks are located at 268 and 354 nm, respectively. The quantum yield (QY) of PEI–Ag NCs is 4.2%. The stability assay demonstrated that the fluorescence can maintain stability for at least 1 month at room temperature. Moreover, the diameters of these particles are mainly distributed in the range 1.6–4.8 nm with an average diameter of 2.95 nm (Additional file 1: Figure S2), illustrating that the PEI–Ag NCs possess the properties of small size and well-dispersed. Thus, the RRS signal of free PEI–Ag NCs is very weak, which makes these particles serve as a useful probe in RRS assay.

### RRS differentiation of telomere length by PEI–Ag NCs

**Fig. 1:**
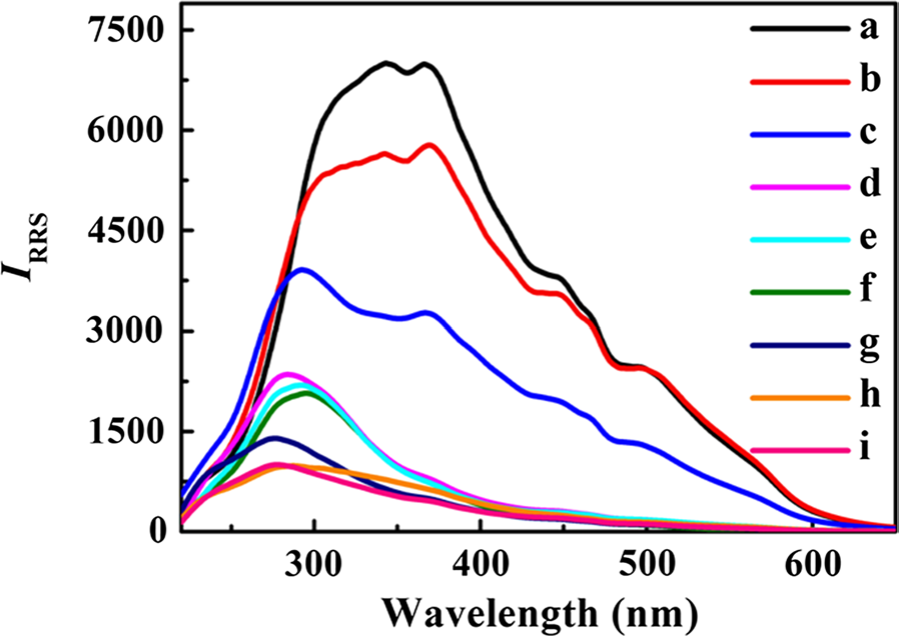
RRS spectra of PEI–Ag NCs/telomere DNA (Tel 10, 22, 40 and 64) system. **a** PEI–Ag NCs/Tel 64, **b** PEI–Ag NCs/Tel 40, **c** PEI–Ag NCs/Tel 22, **d** Tel 64, **e** Tel 40, **f** Tel 22, **g** PEI–Ag NCs/Tel 10, **h** Tel 10, **i** PEI–Ag NCs. The G-rich strands are 30 nM

The RRS spectra of PEI–Ag NCs with addition of G-rich strands (Tel 10, 22, 40 and 64, respectively) are recorded. In Fig. 1, the free PEI–Ag NCs or DNA sequences alone display weak RRS intensities over the range of 220–670 nm; However, in comparison with the blank (free PEI–Ag NCs or DNA alone), the RRS intensities of the solution strongly enhance when PEI–Ag NCs interact with G-rich strand to form complexes. Especially, the enhancement of RRS signals is directly proportional to the length of telomere sequence with the same concentration, and the order is Tel 64 > Tel 40 > Tel 22 > Tel 10. Besides, the same phenomena can also be obtained in the mixture of PEI–Ag NCs and C-rich strands (Ael 10, 22, 40 and 64; Additional file 1: Figure S3). Hence, the RRS method can be applied to discrimination of telomere length in an accurate way.

Notably, although the length of DNA is different, the mechanism of RRS enhancement based on the interaction between PEI–Ag NCs and telomere DNA is same. Hence, Tel 22 and Tel 64 are taken as examples to optimize the experimental conditions for the best assay performance (Additional file 1: Figures S4–S7). The optimal concentration of probe is 0.1 μL mL^−1^; the reaction pH is 7.4 (Tris-HAC buffer); the proper reaction temperature is 4 °C and the reaction can be completed within 2 h. Besides, in order to test the possible repeatability problem induced by the background signal fluctuation due to probe synthesis, several batches of PEI–Ag NCs have been synthesized in 3 different days over a period of 1 week. These probes are utilized to detect Tel 64 (20 nM), and the relative standard deviation (RSD) of interassay (n=3) is calculated in Additional file 1: Table S2. It is found that the RSD of inter-day precision (CV%) is about 2.0–3.9%, suggesting that the error introduced by the probe synthesis can be neglected and this strategy shows a good reproducibility.

### Sensitivity

**Fig. 2:**
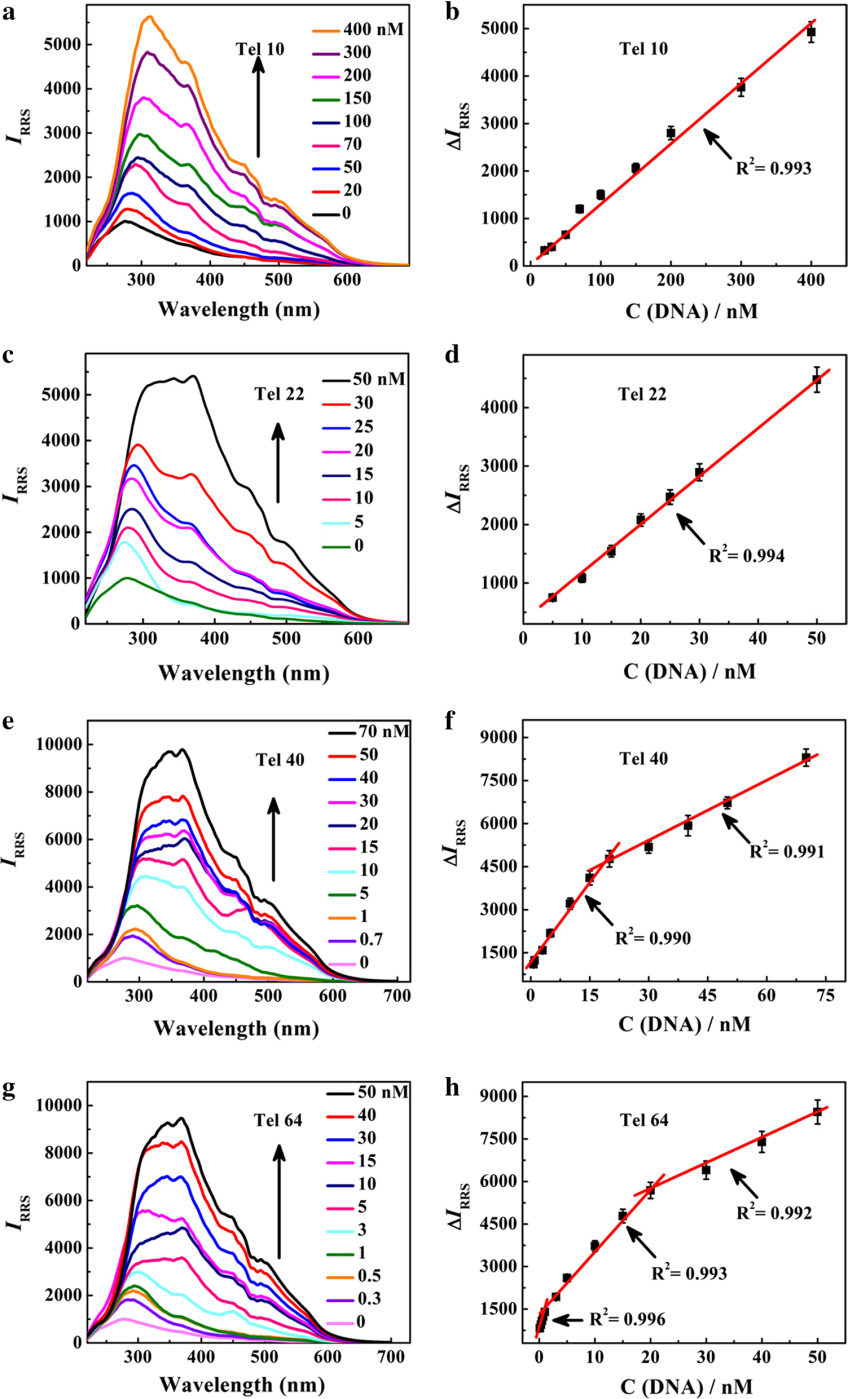
RRS spectra of PEI–Ag NCs upon addition of different concentrations of G-rich strands and the corresponding linear ranges (**a**, **b** Tel 10; **c**, **d** Tel 22; **e**, **f** Tel 40; **g**, **h** Tel 64)

Under the optimized conditions discussed above, the RRS spectra of PEI–Ag NCs are recorded upon the addition of G-rich strands at different amounts. As depicted in Additional file 1: Figure S8, the free Tel 10, Tel 22, Tel 40 and Tel 64 exhibit slight changes of RRS intensities under the large concentrations. In contrast, when PEI–Ag NCs react with different concentrations of G-rich strands, the RRS intensities enhance linearly (Fig. 2) with the concentration range from 20 to 400 nM for Tel 10, 5 to 50 nM for Tel 22, 0.7 to 70 nM for Tel 40 and 0.3 to 50 nM for Tel 64, respectively. The limits of detection (LOD) of Tel 10, Tel 22, Tel 40 and Tel 64 are estimated (3σ/S, σ was the standard deviation of the blank solution) to be 6.73, 0.97, 0.42 and 0.12 nM, respectively. The RRS method can also be used to recognize the length of C-rich strand; the corresponding RRS spectra and calibration curves of PEI–Ag NCs with the addition of Ael 10, Ael 22, Ael 40 and Ael 64 are displayed in Additional file 1: Figure S9 and S10. In comparison with the corresponding linear ranges and LODs of telomere DNA (Additional file 1: Table S3), for both G-rich strands and C-rich strands, the sensitivity of 64 bases is the highest, while that of 10 bases is the lowest, thus PEI–Ag NCs exhibit higher sensitivity for long telomere DNA and can discriminate the length of DNA substrates.

### Mechanism of RRS enhancement

Three reasons may explain the RRS enhancement in the mixture of PEI–Ag NCs and telomere DNA:

#### (1) Increase of the scattering molecular volume

It is proverbial that increasing the volume of scattering molecule is advantageous to the enhancement of scattering intensity [34]. DNA is a biopolymer composed of building blocks called nucleotides consisting of deoxyribose sugar, a phosphate group and side group amine bases [35]. Due to the presence of phosphate group, DNA is characterized by abundant moieties of negative charge [36]. However, in dilute aqueous solution, the PEI–Ag NCs exist as positively charged clusters (ζ = 155 mV). Thus, PEI–Ag NCs and DNA can interact with each other via electrostatic attraction, leading to the augment of the molecular volume. According to the Rayleigh scattering formula [37], I_RRS_ = KCMI_0_, where I_RRS_ is the resonance Rayleigh scattering intensity, K is a constant, C is the concentration of the scattering molecules, M is the molecular weight and I_0_ is the incident light intensity. When I_0_ and C are constant, the intensity of the I_RRS_ is directly proportional to the molecular weight of the scattering particle. Hence, the formation of complexes with a large volume is an important contributor to the observed scattering enhancement. In addition, the longer the length of telomere DNA is, the stronger the reflection intensity can be obtained. Therefore, according to different degrees of enhancement of RRS signals, the discrimination of telomere length can be actualized at the condition of these DNA sequences with the same concentration.

#### (2) Enhancement of hydrophobicity

The hydrophobic interfaces are another reason for a surface-enhanced scattering effect [38]. The PEI–Ag NCs are positively charged (ζ = 155 mV), while the ζ potential of PEI–Ag NCs and DNA complexes are 22 mV. Therefore, when PEI–Ag NCs and DNA interact with each other to form combined products, the negative charges of telomere DNA and the positive charges of PEI–Ag NCs are neutralized to a large extent, leading to the hydrophobicity enhancement due to the electroneutrality state and the hydrophobic moiety of the ion-association [39]. Thus, RRS signals obviously increase because of the strongly hydrophobic interaction.

#### (3) Resonance enhanced effect

**Fig. 3:**
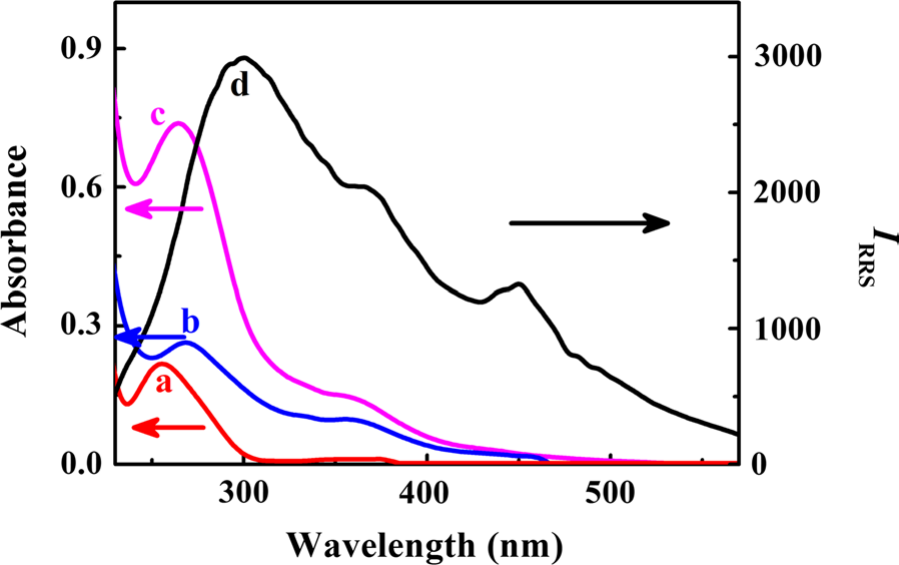
Comparison of absorption spectra (**a** Tel 64; **b** PEI–Ag NCs; **c** PEI–Ag NCs/Tel 64) and RRS spectrum (**d** PEI–Ag NCs/Tel 64)

Resonance Rayleigh scattering is an absorption-rescattering process produced by the resonance between the Rayleigh scattering and the light absorption with identical frequency. When the wavelength of Rayleigh is located at or closed to the absorption band, the scattering intensity is strengthened by several orders of magnitude than a single Rayleigh scattering [27]. Taking Tel 64 as an example (Fig. 3), the free DNA displays a characteristic peak at 260 nm and the characteristic absorption peaks of PEI–Ag NCs alone appear at 268 and 354 nm. The absorbance rises obviously when the complex of PEI–Ag NCs and Tel 64 forms and the scattering band is located in this absorption band, bringing about a remarkable RRS intensity.

Hence, the combination of the above three factors produces the obvious increase of RRS signal by the formation of complexes between PEI–Ag NCs and telomere DNA.

## Control experiment

### The roles of PEI and Ag nucleus

In the control experiment, the roles of PEI and Ag nucleus are determined through instead of PEI–Ag NCs with free PEI in the reaction with DNA. In Additional file 1: Figure S11, the weak RRS signals of free PEI and Tel 64 alone are obtained; when PEI reacts with Tel 64 to form combined products, the intensity of RRS also increases and the degree of enhancement is lower than that of PEI–Ag NCs and DNA mixture at the same concentration, suggesting that free PEI can also react with telomere DNA via electrostatic attraction; however, Ag nucleus, as an electron-deficient acceptor [40, 41], can interact with DNA sequences which have a lot of electron-rich groups, such as hydroxyl, phosphoric acid groups and carboxyl groups. Therefore, both PEI and Ag nucleus play important roles to generate a stronger RRS signal in the interaction with DNA.

### Differentiation of other DNA lengths by PEI–Ag NCs

This RRS strategy is utilized to discrimination of other DNA lengths (PSM.2 and HIV). The RRS intensities of free DNA (PSM.2 and HIV) are very weak (Additional file 1: Figure S12). By contrast, the intensities of RRS of PEI–Ag NCs/DNA gradually rise as the DNA concentrations increase (Additional file 1: Figure S13). Good linear relationships are found for PSM.2 and HIV concentrations varying from 3 to 50 nM and 3 to 70 nM, respectively. The result is strengthened by the fact that PEI–Ag NCs is a useful probe to distinguish DNA length. When the number of nucleotides of two DNA sequence differs by more than 7 (Additional file 1: Figure S14), this strategy can successfully distinguish the length of DNA based on the differences of RRS data, such as HIV (18 bases) and Tel 40 (40 bases). Furthermore, the enhancement of RRS intensity of PSM.2 (18 bases) is close to that of Tel 22 (22 bases) suggesting that the PEI–Ag NCs can not recognize them because of the approximate base number (4 bases). The evaluation of telomere length has greater significance in understanding of human longevity, so this RRS method may be an original and useful for the detection of telomere DNA length.

## Construction of a logic gate

**Scheme 1:**
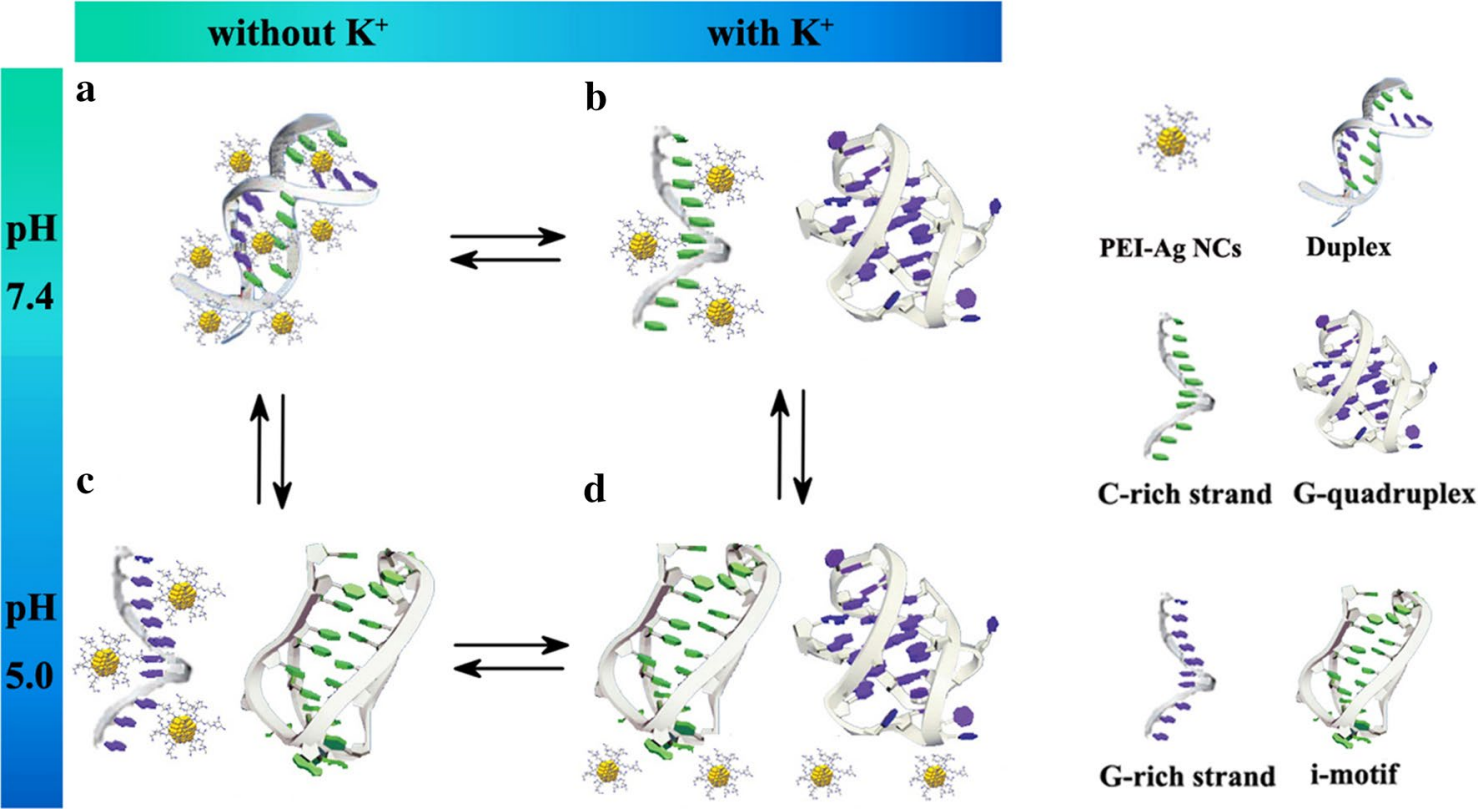
Schematic illustration of structural conversion of the 1:1 mixture of G and C strands among duplex, quadruplex, and random coil forms as controlled by K^+^ and H^+^ (**a** duplex; **b** G-quadruplex and C random coil; **c** i-motif and G random coil; **d** G-quadruplex and i-motif)

**Fig. 4:**
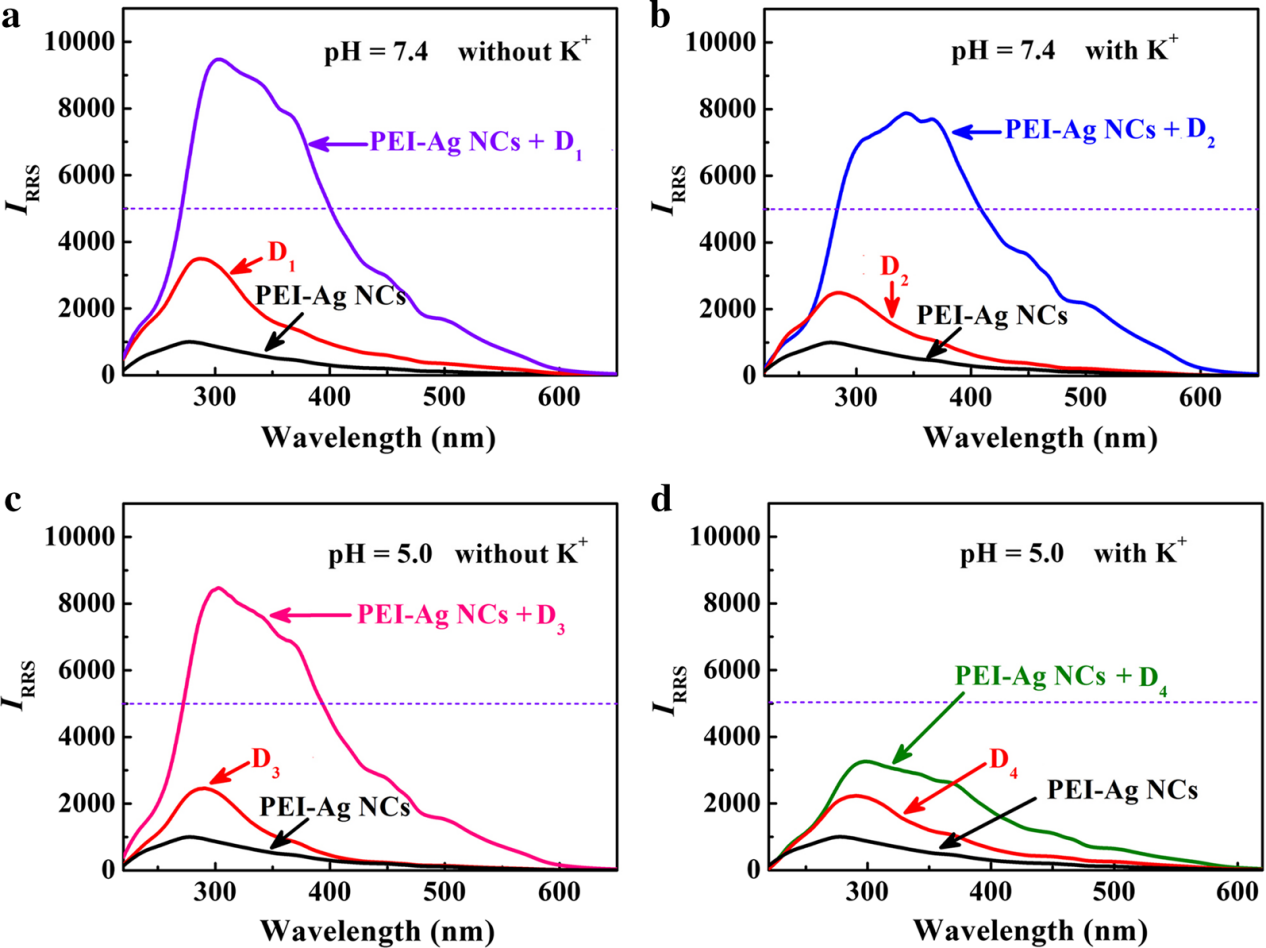
RRS spectra of PEI–Ag NCs, DNA and PEI–Ag NCs/DNA system in different environments (**a** D1, duplex; **b** D2, G-quadruplex and C random coil; **c** D3, i-motif and G random coil; **d** D4, G-quadruplex and i-motif). The total concentration of DNA is 20 nM

**Fig. 5:**
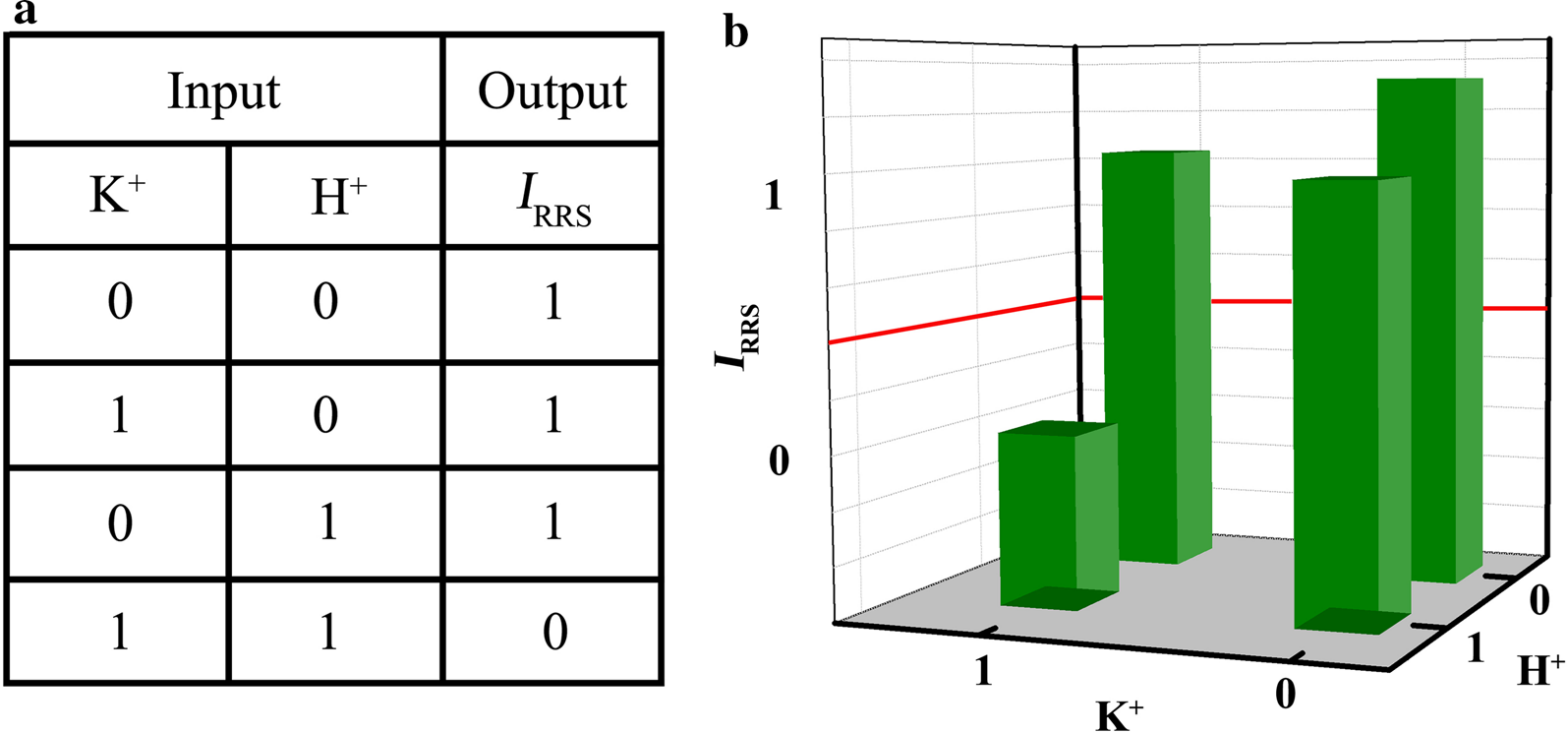
The truth table and design of the NAND logic gate (**a**) and RRS intensities of the mixture of PEI–Ag NCs/telomere DNA in the presence of different inputs, with a threshold of *I*_RRS_= 5000 for output 1 or 0 (**b**)

Another highlight of this assay is that the RRS strategy can recognize specific motifs (G-quadruplex or i-motif) of DNA. When PEI–Ag NCs react with G-quadruplex or i-motif, the intensities of RRS exhibit slight changes (Additional file 1: Figure S15). Moreover, these special motifs are confirmed by CD spectra (Additional file 1: Figure S16). Hence, through two chemical input signals (K^+^ and H^+^) and changes in RRS intensity (output signal), a new concept for a DNA logic gate (NAND) is constructed. Due to the excellent sensitivity of RRS method, the threshold value of RRS intensity at the output is set to 5000, which is about fivefolds higher than that of probe. The DNA logic gate displays four states (Scheme 1, Figs. 4 and 5): (1) in the absence of K^+^ at pH 7.4 (0,0), C-rich strands and G-rich strands form double-stranded, resulting in the maximum enhancement of RRS intensity (Additional file 1: Figure S17 and S18) due to the reaction between PEI–Ag NCs and duplexes (output 1); (2) In the presence of K^+^ at pH 7.4 (1,0), G-rich strand can fold into G quadruplexe and C-rich strand exists predominantly in a random coil conformation, producing evident enhancement of RRS intensity and an output signal of 1; (3) In acidity (pH=5.0) without K^+^ (0,1), C-rich strand fabricates i-motif, while G-rich strand retains random coil, also generating a significantly enhanced signal (output 1); (4) In the presence of K^+^ at pH 5.0 (1,1), the telomere DNA folding into G-quadruplexe and i-motif, the RRS intensity exhibits little change and the output signal is 0.

According to reported literature, the relative scattering intensities of the elongated chain state molecule is greater than that of random coil state, and followed by that of the compact state, like globule state [42]. Thus, in comparison with the single-stranded DNA and special motifs (G-quadruplexe or i-motif or both of G-quadruplexe and i-motif), the telomere DNA double helix structure has a certain degree of deformation and becomes looser, making the PEI–Ag NCs combine with DNA more easily and producing the most obvious enhancement of RRS signals. In contrast, when nucleic acid exists as G-quadruplexe or i-motif, the structures of special motifs are more compacting and difficultly react with PEI–Ag NCs, resulting in slight change of RRS. Apparently, when one strand exists as the extended coil state and the other strand forms a special motif (G-rich strand/i-motif or C-rich strand/G- quadruplexe), the random coil can also interact with PEI–Ag NCs to form complexes, producing the enhancement of RRS intensity. Furthermore, compared with previously published DNA logic gate involving labeled-DNA [21], this logic gate operation is label-free and consuming less DNA, so ours is more green, low-cost, sensitive and efficient.

## Conclusions

Herein, the PEI–Ag NCs can serve as a novel RRS probe to identify DNA length and monitor G-quadruplex/i-motif through the different increasing degrees of RRS intensity, especially for i-motif, which does not attract much attention for its detection and recognition. Furthermore, the RRS signal of PEI–Ag NCs also varies with the structure conversion of telomere DNA molecules among multiple surrounding conditions. Therefore, a simple and robust DNA logic gate (NAND) is established using K^+^ and H^+^ as the two inputs and changes in RRS intensity as the output signal. Comparing with the our previously reported colorimetric method for differentiating telomere DNA [43], this work shows more advantages, including the high sensitivity, simple operation, low cost, and good reproducibility. We believe that this work may shed some light for identifying DNA length and monitoring special motifs.

## Additional file

**Additional file 1: Figure S1.** UV-vis absorption and fluorescence spectra of PEI-Ag NCs. **Figure S2.** TEM images of PEI-Ag-NCs. **Figure S3.** RRS spectra of PEI-Ag NCs/telomere DNA (C-rich strands, Ael 10, 22, 40 and 64) system. **Figure S4.** Influence of PEI-Ag NCs concentration on the detection of telomere DNA. **Figure S5.** Influence of pH values on the detection of Tel 22 and Tel 64. **Figure S6.** Influence of reaction temperature on the detection of Tel 22 and Tel 64. **Figure S7.** Influence of reaction time on the detection of Tel 22 and Tel 64. **Figure S8.** RRS signals of free G-rich strands with different concentrations**. Figure S9.** RRS spectra of PEI-Ag NCs upon addition of different concentrations of C-rich strands and the corresponding linear ranges. **Figure S10.** RRS signals of free C-rich strands with different concentrations. **Figure S11.** Comparison of RRS spectra of PEI-Ag NCs/Tel 64 and PEI/Tel 64. **Figure S12.** RRS signals of free DNA (PSM.2 and HIV) with different concentrations. **Figure S13.** RRS spectra of PEI-Ag NCs upon addition of different concentrations of DNA (PSM.2 and HIV) and the corresponding linear ranges. **Figure S14.** Comparison of RRS signals of different PEI-Ag NCs/DNA system (Tel 10, PSM.2, Tel 22, HIV, Tel 40 and Tel 64). **Figure S15.** Comparison of RRS spectra of PEI-Ag NCs, motifs and PEI-Ag NCs/motif system. **Figure S16.** CD spectra of G-rich strands and C-rich strands in the presence or absence of K^+^ or H^+^. **Figure S17.** RRS spectra of PEI-Ag NCs, DNA and PEI-Ag NCs/DNA system. **Figure S18.** The RRS signals of DNA in absence and presence of PEI-Ag NCs. **Table S1.** Oligonucleotides used in this work. **Table S2.** Reproducibility of RRS method. **Table S3.** Linear ranges and correlation coefficients of the calibration graphs, and the detection limits for telomere DNA.

## Abbreviations

PEI: polyethyleneimine
PEI–Ag NCs: Ag nanoclusters templated by polyethyleneimine
RRS: resonance Rayleigh scattering
UV–vis: ultraviolet–visible
AgNO_3_: silver nitrate
HCHO: formaldehyde
HAC: acetic acid
Tris: trihydroxymethyl aminomethane
NaAC: sodium acetate
KAC: potassium acetate
CD: circular dichroism
LOD: limits of detection
