# Supplementary material for: A resonance Rayleigh scattering sensor for sensitive differentiation of telomere DNA length and monitoring special motifs (G-quadruplex and i-motif) based on the Ag nanoclusters and NAND logic gate responding to chemical input signals
Source: J Nanobiotechnology. 2018 Oct 9;16:78. doi: 10.1186/s12951-018-0407-5 (PMC6176526; doi:10.1186/s12951-018-0407-5)
Supplement: Supplementary file 1 — Additional file 1: Figure S1. UV-vis absorption and fluorescence spectra of PEI-Ag NCs. Figure S2. TEM images of PEI-Ag-NCs. Figure S3. RRS spectra of PEI-Ag NCs/telomere DNA (C-rich strands, Ael 10, 22, 40 and 64) system. Figure S4. Influence of PEI-Ag NCs concentration on the detection of telomere DNA. Figure S5. Influence of pH values on the detection of Tel 22 and Tel 64. Figure S6. Influence of reaction temperature on the detection of Tel 22 and Tel 64. Figure S7. Influence of reaction time on the detection of Tel 22 and Tel 64. Figure S8. RRS signals of free G-rich strands with different concentrations. Figure S9. RRS spectra of PEI-Ag NCs upon addition of different concentrations of C-rich strands and the corresponding linear ranges. Figure S10. RRS signals of free C-rich strands with different concentrations. Figure S11. Comparison of RRS spectra of PEI-Ag NCs/Tel 64 and PEI/Tel 64. Figure S12. RRS signals of free DNA (PSM.2 and HIV) with different concentrations. Figure S13. RRS spectra of PEI-Ag NCs upon addition of different concentrations of DNA (PSM.2 and HIV) and the corresponding linear ranges. Figure S14. Comparison of RRS signals of different PEI-Ag NCs/DNA system (Tel 10, PSM.2, Tel 22, HIV, Tel 40 and Tel 64). Figure S15. Comparison of RRS spectra of PEI-Ag NCs, motifs and PEI-Ag NCs/motif system. Figure S16. CD spectra of G-rich strands and C-rich strands in the presence or absence of K+ or H+. Figure S17. RRS spectra of PEI-Ag NCs, DNA and PEI-Ag NCs/DNA system. Figure S18. The RRS signals of DNA in absence and presence of PEI-Ag NCs. Table S1. Oligonucleotides used in this work. Table S2. Reproducibility of RRS method. Table S3. Linear ranges and correlation coefficients of the calibration graphs, and the detection limits for telomere DNA. [file 12951_2018_407_MOESM1_ESM.doc]

**Additional Information for**

**A Resonance Rayleigh Scattering sensor for sensitive differentiation of telomere DNA length and** **monitoring special motifs (G-quadruplex and i-motif)** **based on the Ag nanoclusters and NAND** **logic gate responding to chemical input signals**

Shuai Wang^a,b^, Fei Qu^a*^, Wenli Han^c^, Jinmao You^a,d^

^a^ The Key Laboratory of Life-Organic Analysis, Qufu Normal University, Qufu 273165, Shandong, China

^b^ The Key Laboratory for Colloid and Interface Chemistry of Education Ministry, Shandong University, Jinan 250100, Shandong, China

^c^ Laboratory Animal Center, Chongqing Medical University, Chongqing, China

^d^ Northwest Institute of Plateau Biology, Chinese Academy of Sciences, Xining 810001, China

**Corresponding Authors**

^*^ E-mail: qufei3323@163.com (F. Qu)

**
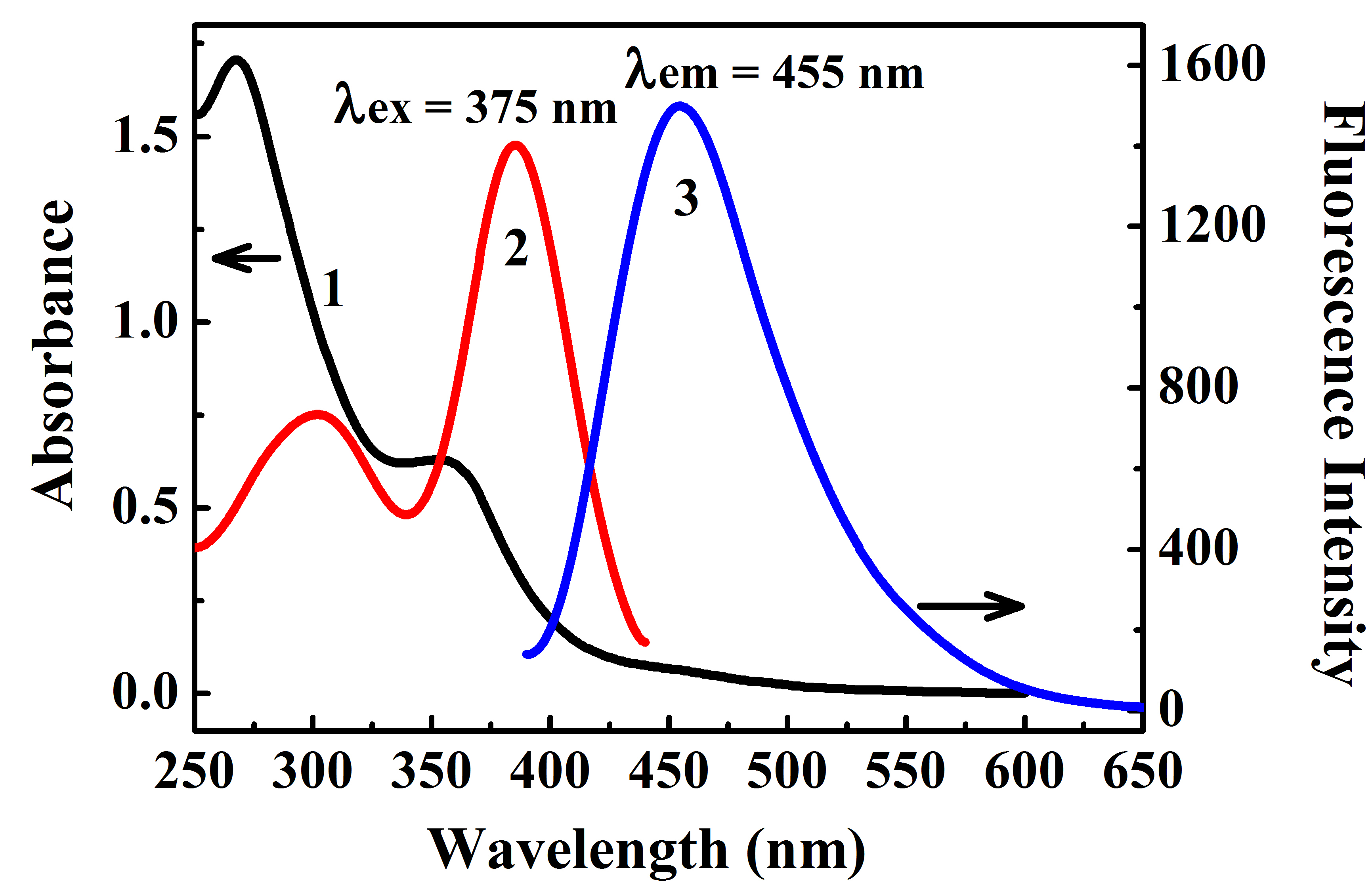
**

**Figure S1** UV-vis absorption and fluorescence spectra of PEI-Ag NCs.


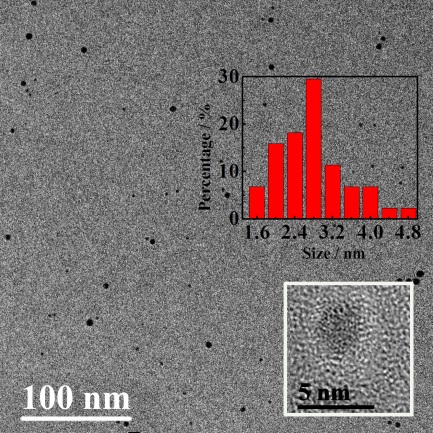


**Figure S2** TEM images of PEI-Ag-NCs.


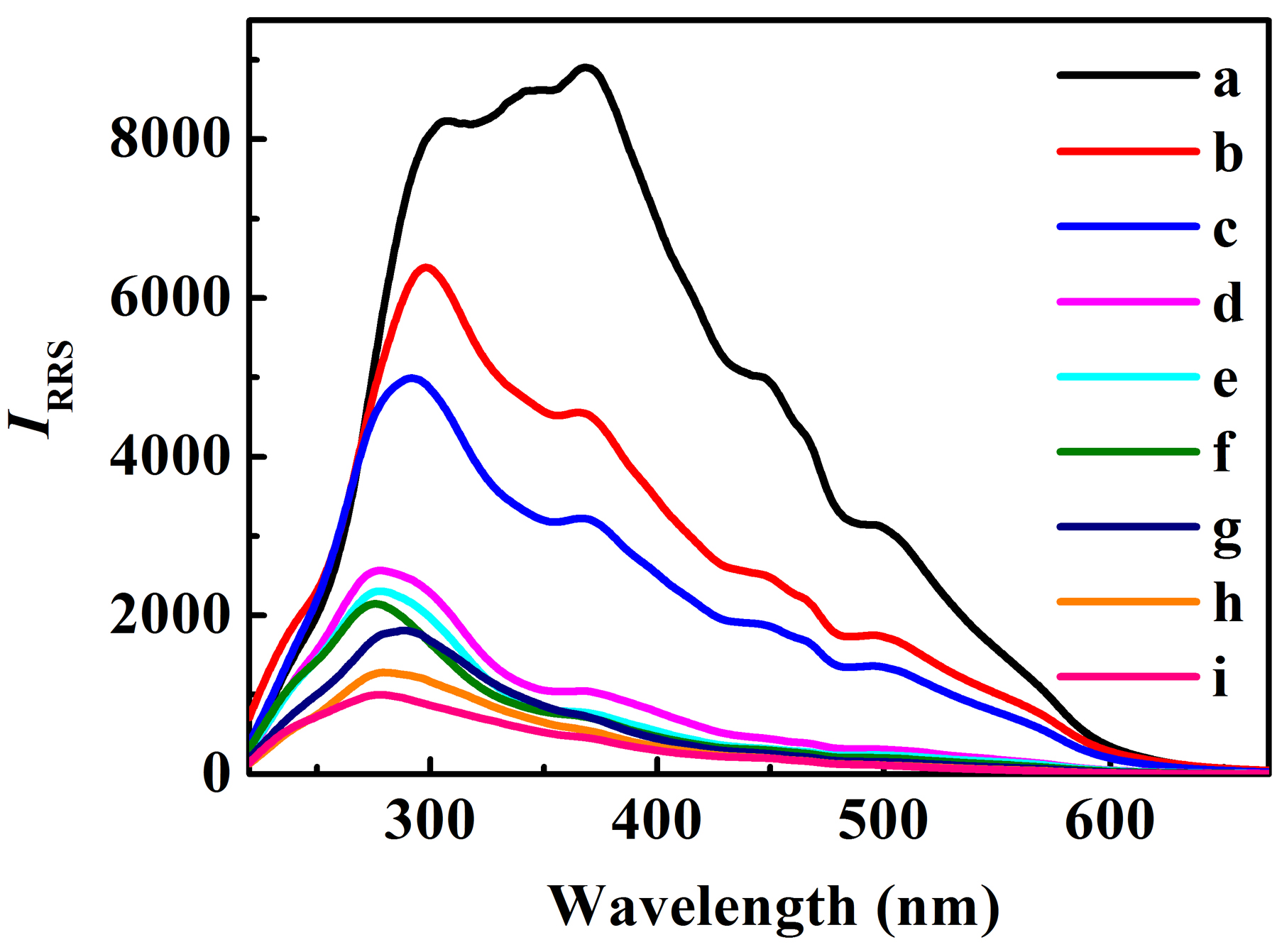


**Figure S3** RRS spectra of PEI-Ag NCs/telomere DNA (C-rich strands, Ael 10, 22, 40 and 64) system. (a) PEI-Ag NCs/Ael 64, (b) PEI-Ag NCs/Ael 40, (c) PEI-Ag NCs/Ael 22, (d) Ael 64, (e) Ael 40, (f) Ael 22, (g) PEI-Ag NCs/Ael 10, (h) Ael 10, (i) PEI-Ag NCs. The C-rich strands are 30 nM.


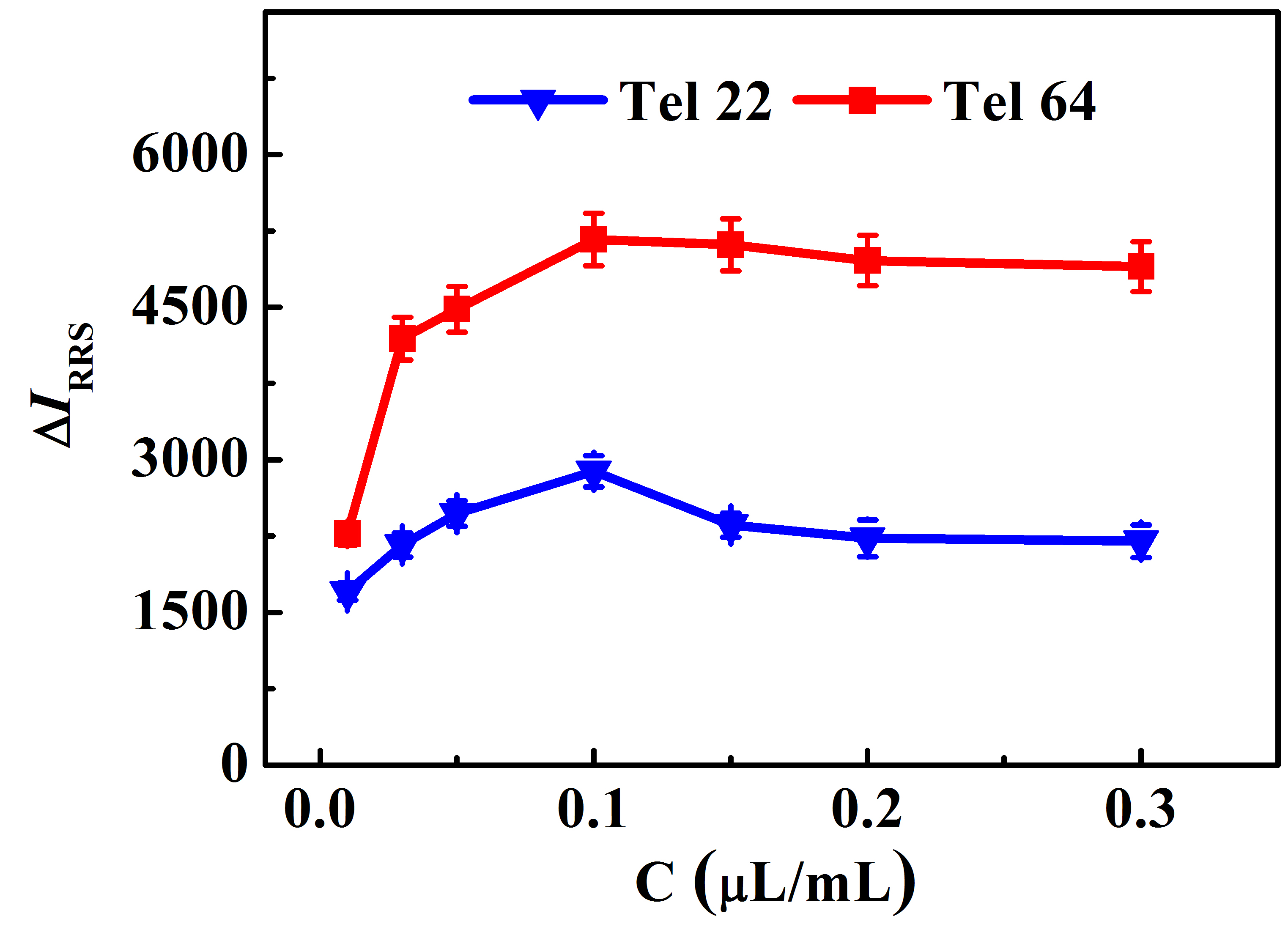


**Figure S4** Influence of PEI-Ag NC concentration on the detection of telomere DNA. The concentrations of Tel 22 and Tel 64 are 20 nM.


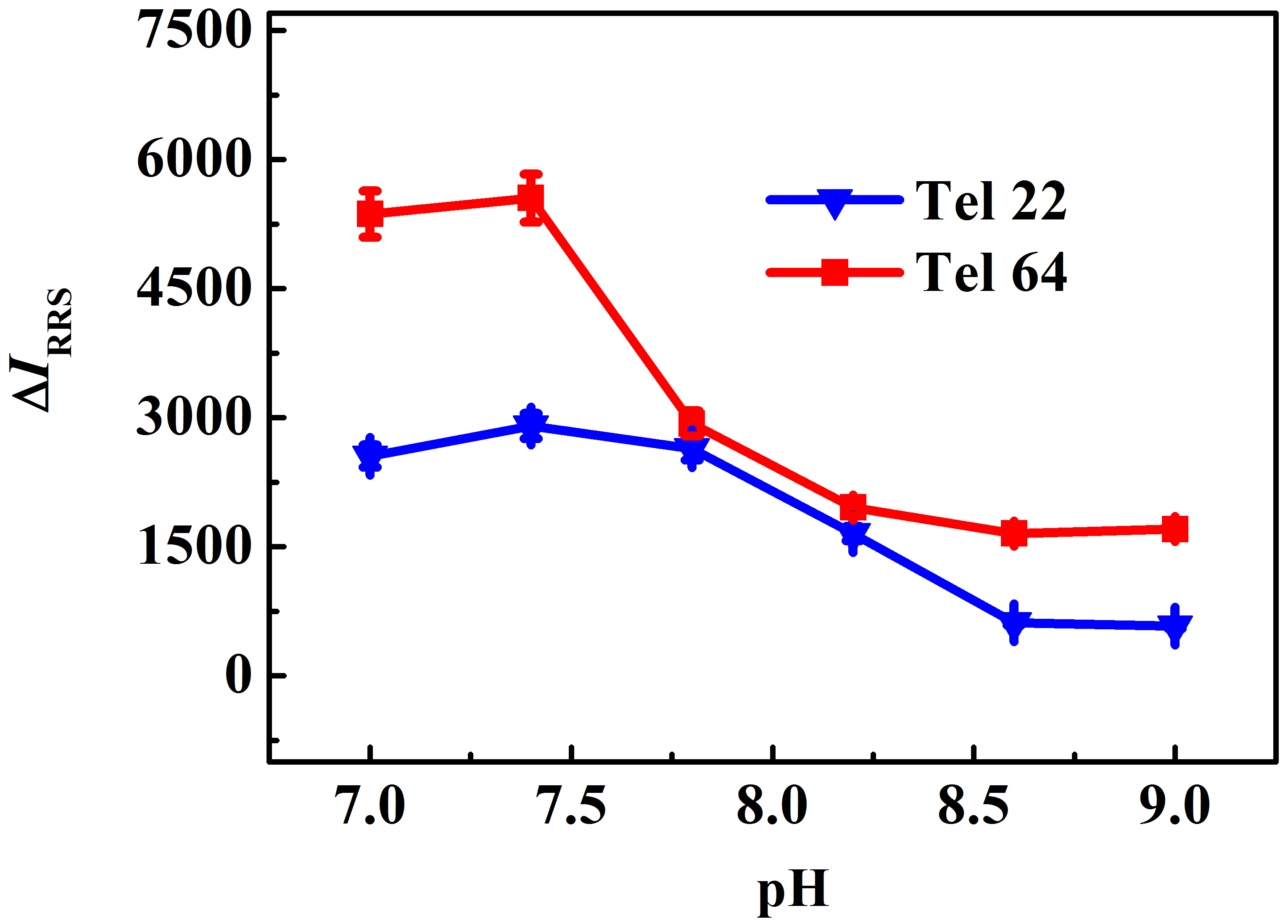


**Figure S5** Influence of pH values on the detection of Tel 22 and Tel 64. The concentrations of Tel 22 and Tel 64 are 20 nM in 10 mM Tris-HAC buffer.


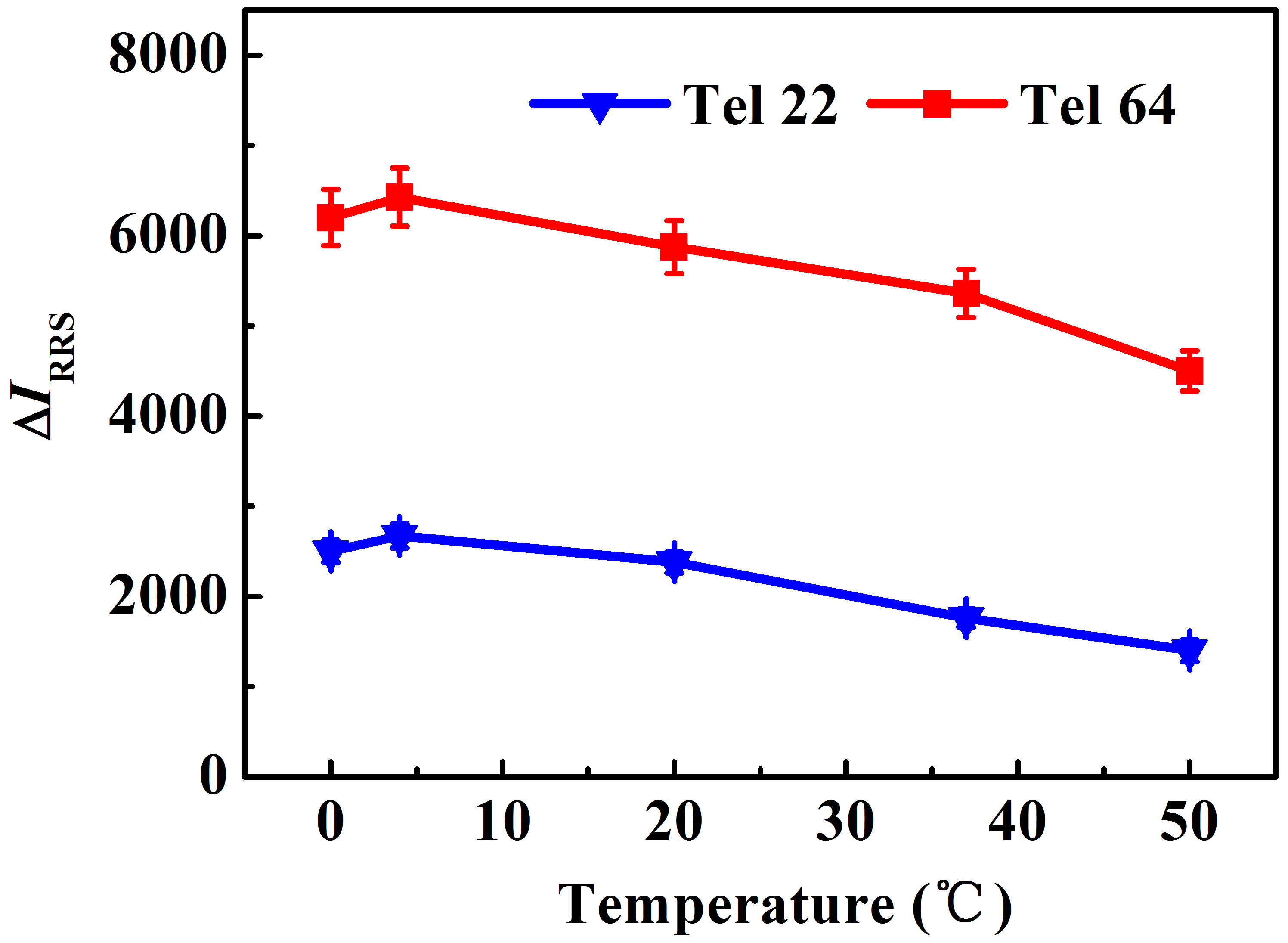


**Figure S6** Influence of reaction temperature on the detection of Tel 22 and Tel 64. The concentrations of Tel 22 and Tel 64 are 20 nM.


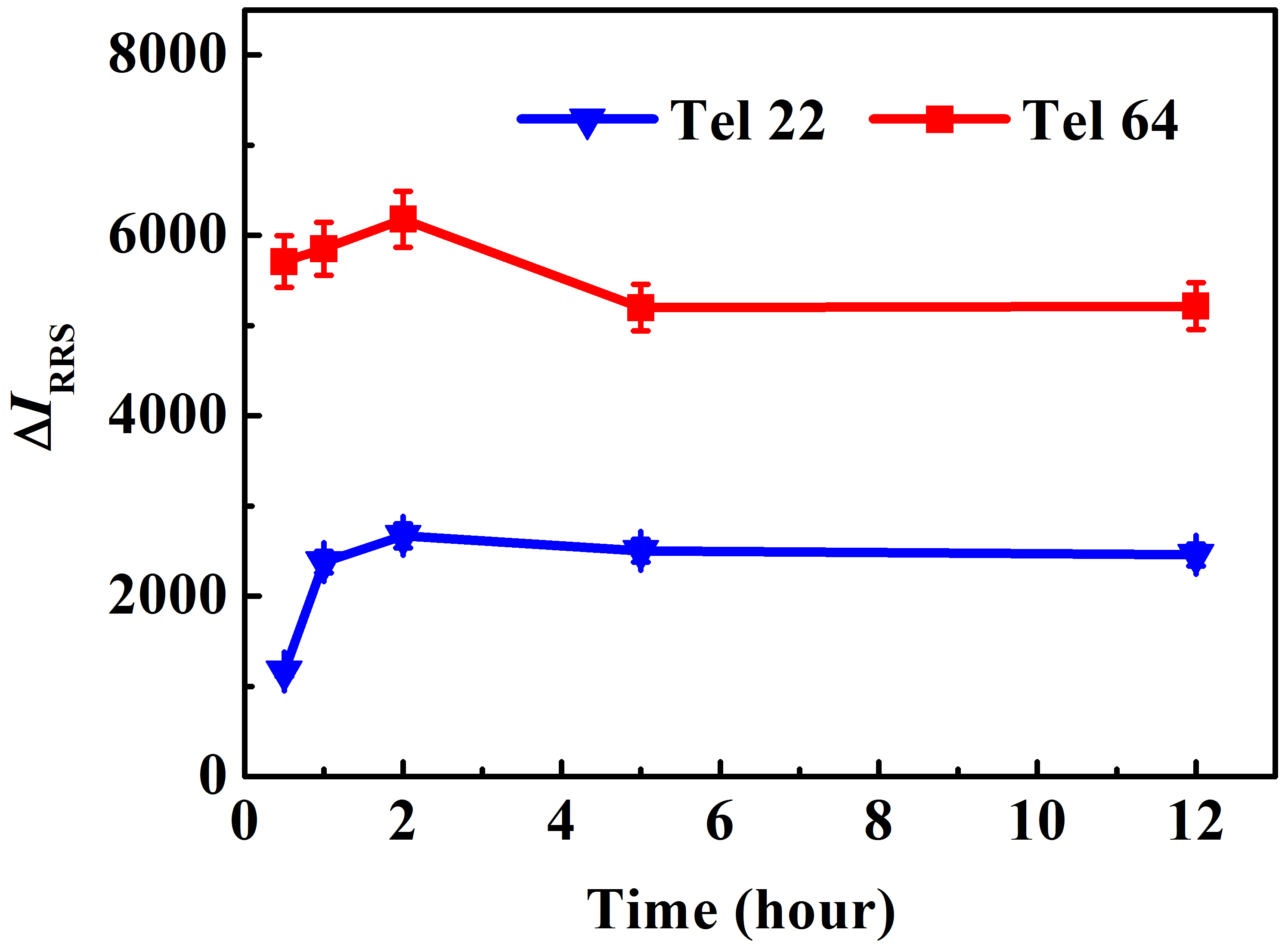


**Figure S7** Influence of reaction time on the detection of Tel 22 and Tel 64. The concentrations of Tel 22 and Tel 64 are 20 nM.


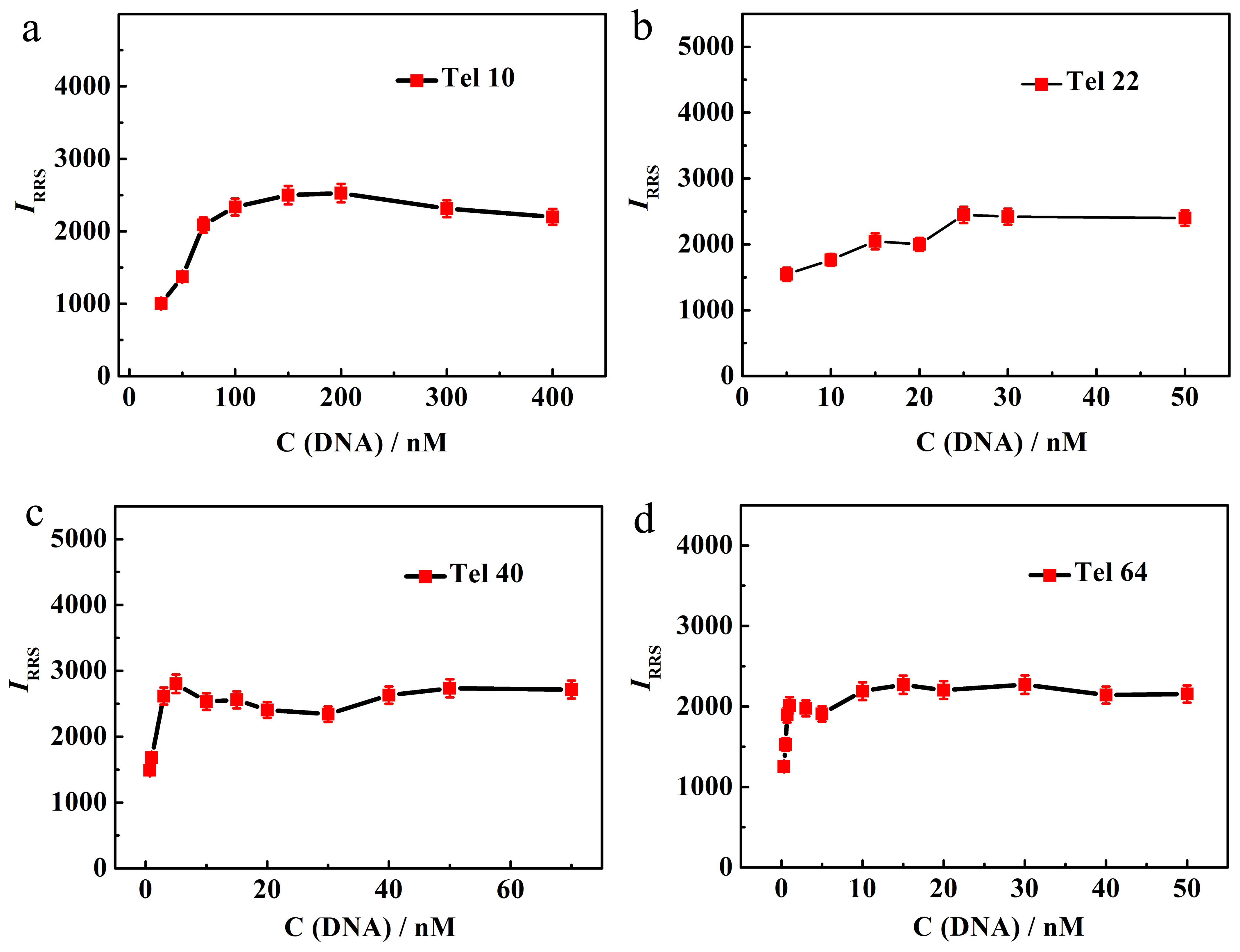


**Figure S8** RRS signals of free G-rich strands with different concentrations (a, Tel 10; b, Tel 22; c, Tel 40; d, Tel 64).


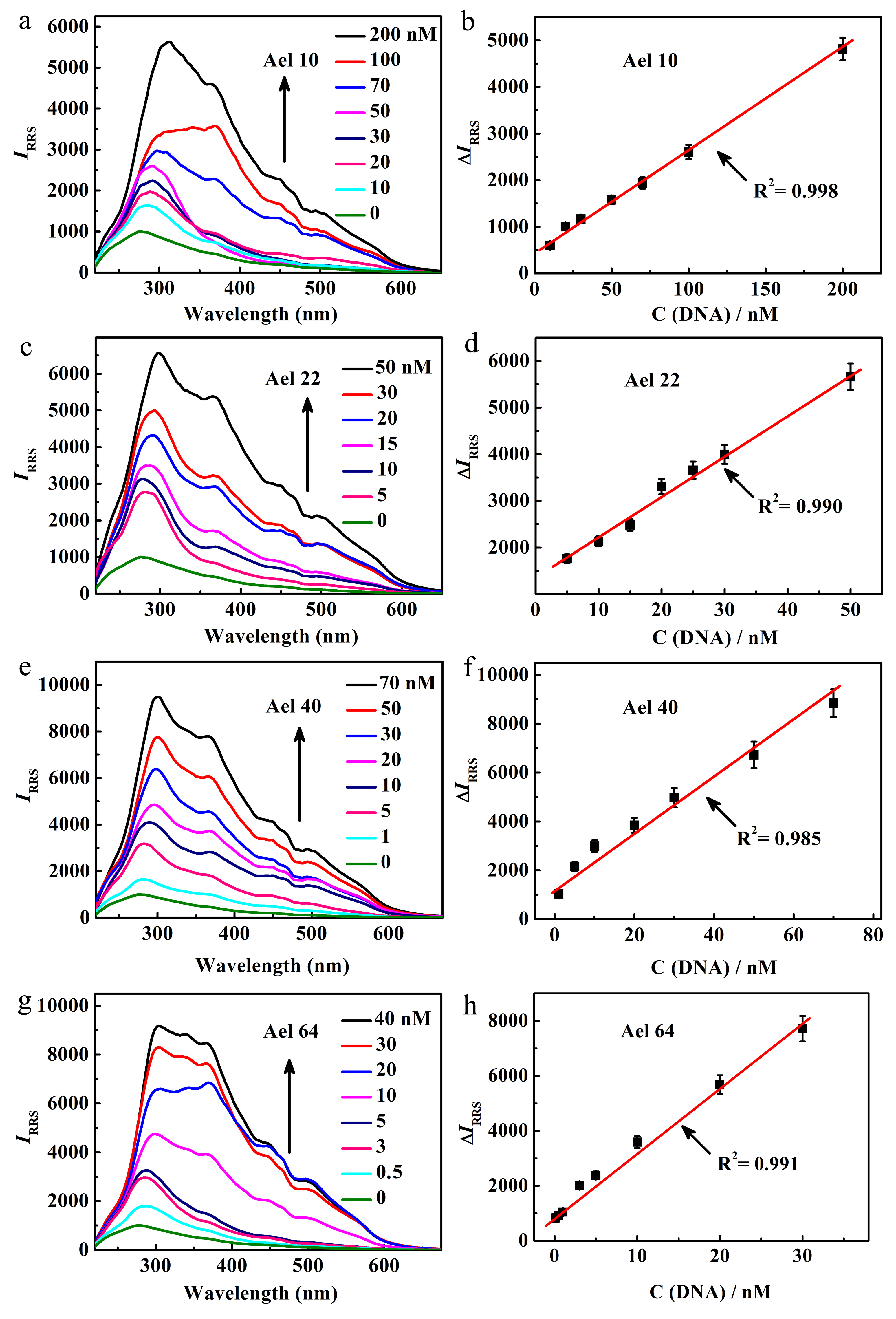


**Figure S9** RRS spectra of PEI-Ag NCs upon addition of different concentrations of C-rich strands and the corresponding linear ranges (a, b, Ael 10; c, d, Ael 22; e, f, Ael 40; g, h, Ael 64).


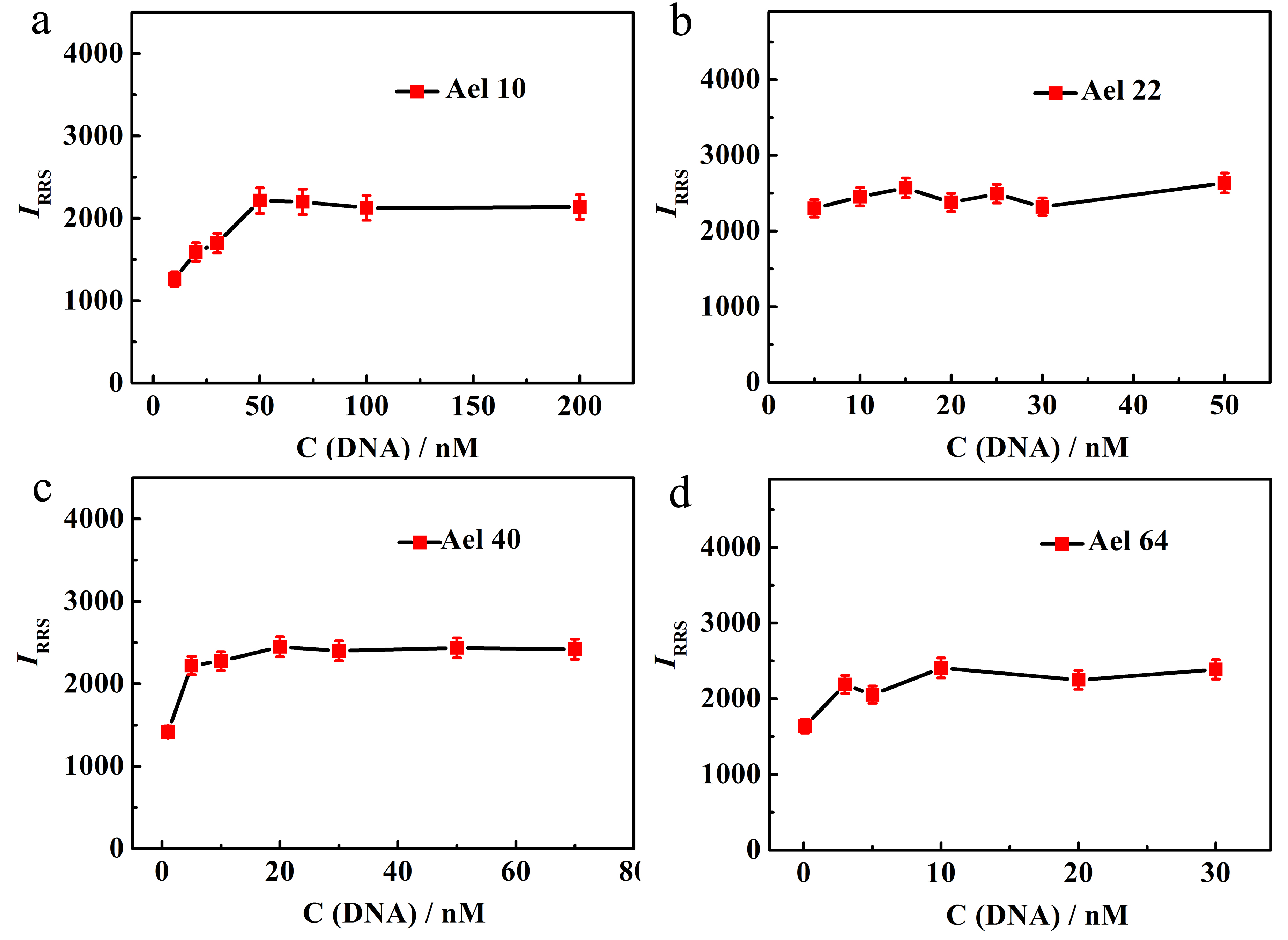


**Figure S10** RRS signals of free C-rich strands with different concentrations (a, Ael 10; b, Ael 22; c, Ael 40; d, Ael 64).

**
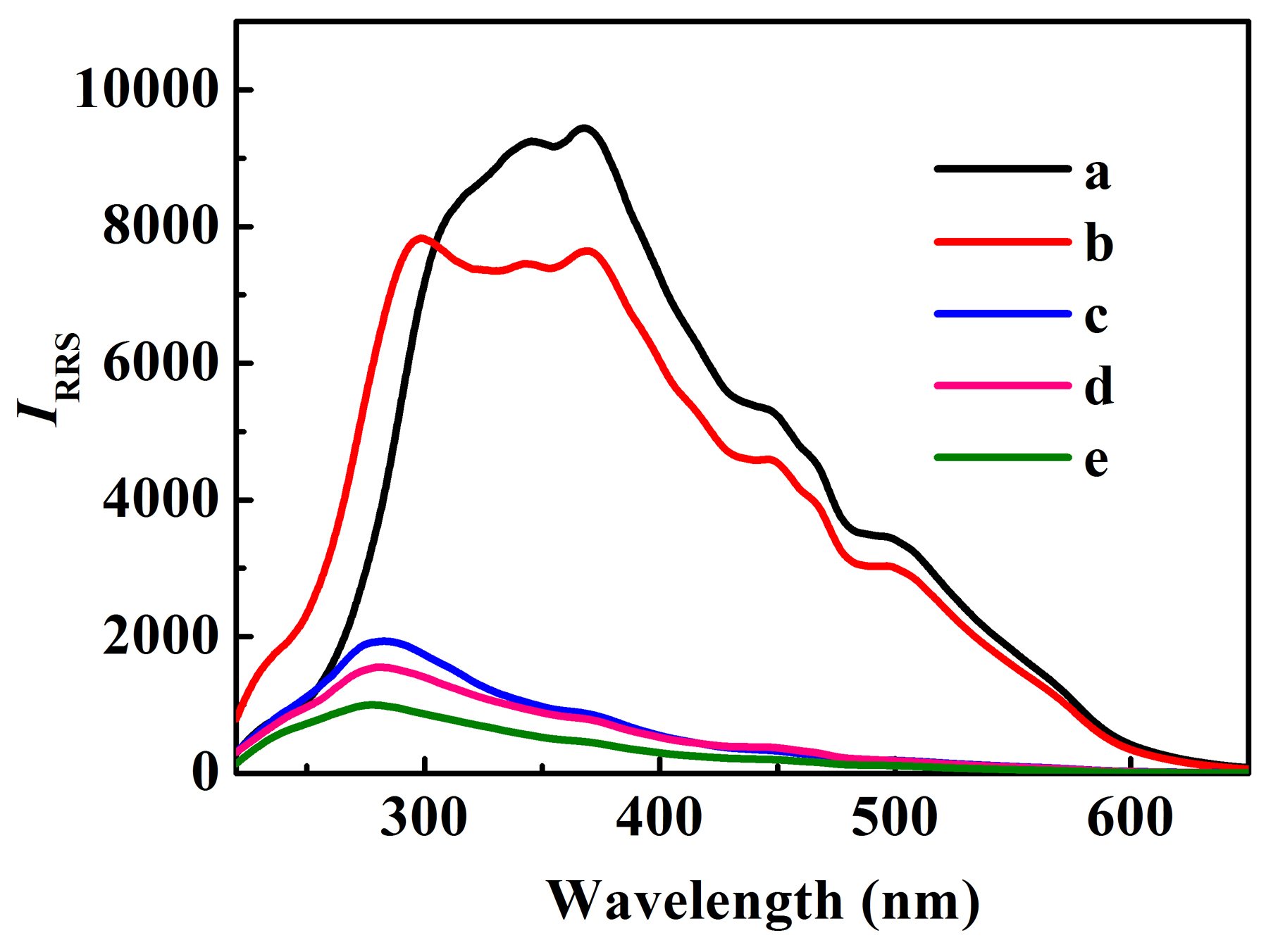
**

**Figure S11** Comparison of RRS spectra of PEI-Ag NCs/Tel 64 and PEI/Tel 64. (a) PEI-Ag NCs/Tel 64 (b) PEI/Tel 64, (c) Tel 64, (d) PEI, (e) PEI-Ag NCs. The concentration of Tel 64 is 50 nM.


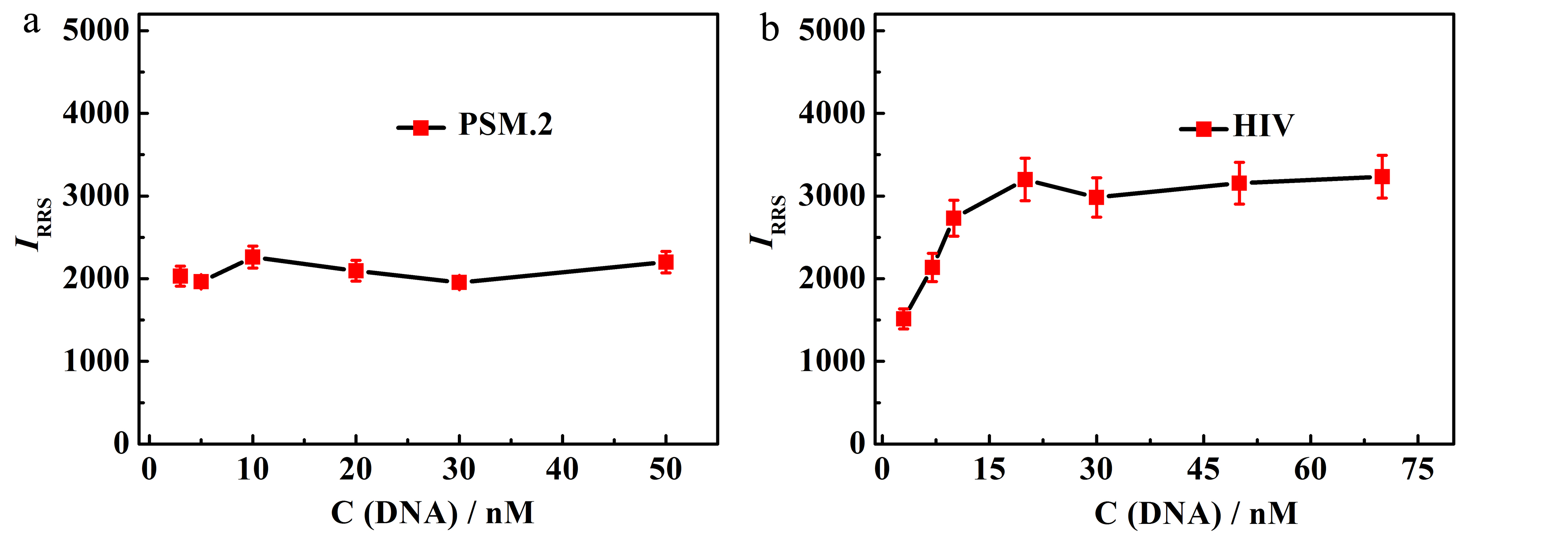


**Figure S1****2** RRS signals of free DNA with different concentrations (a, PSM.2; b, HIV).


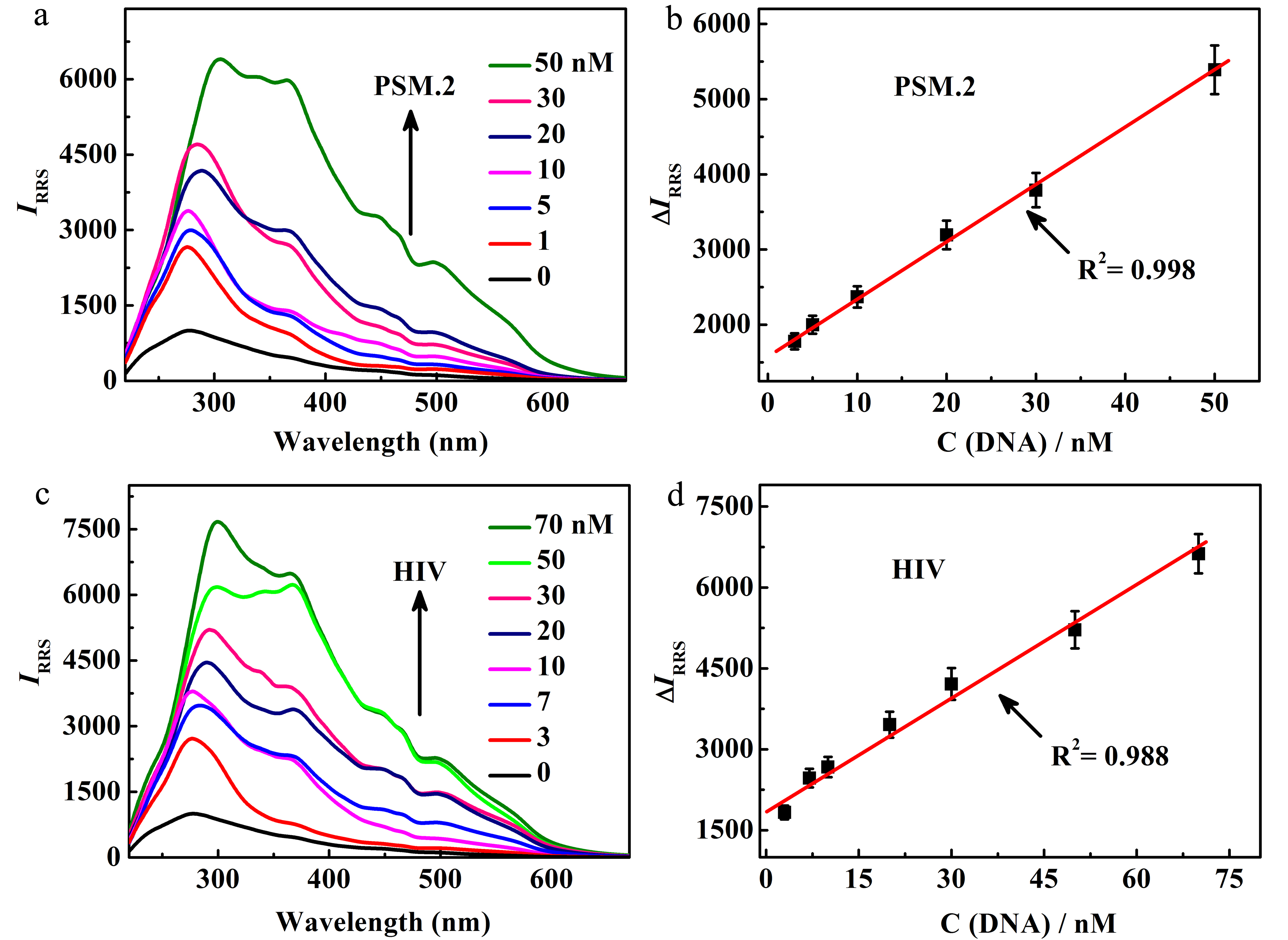


**Figure S13** RRS spectra of PEI-Ag NCs upon addition of different concentrations of DNA (PSM.2 and HIV) and the corresponding linear ranges (a, b, PSM.2; c, d, HIV).

**
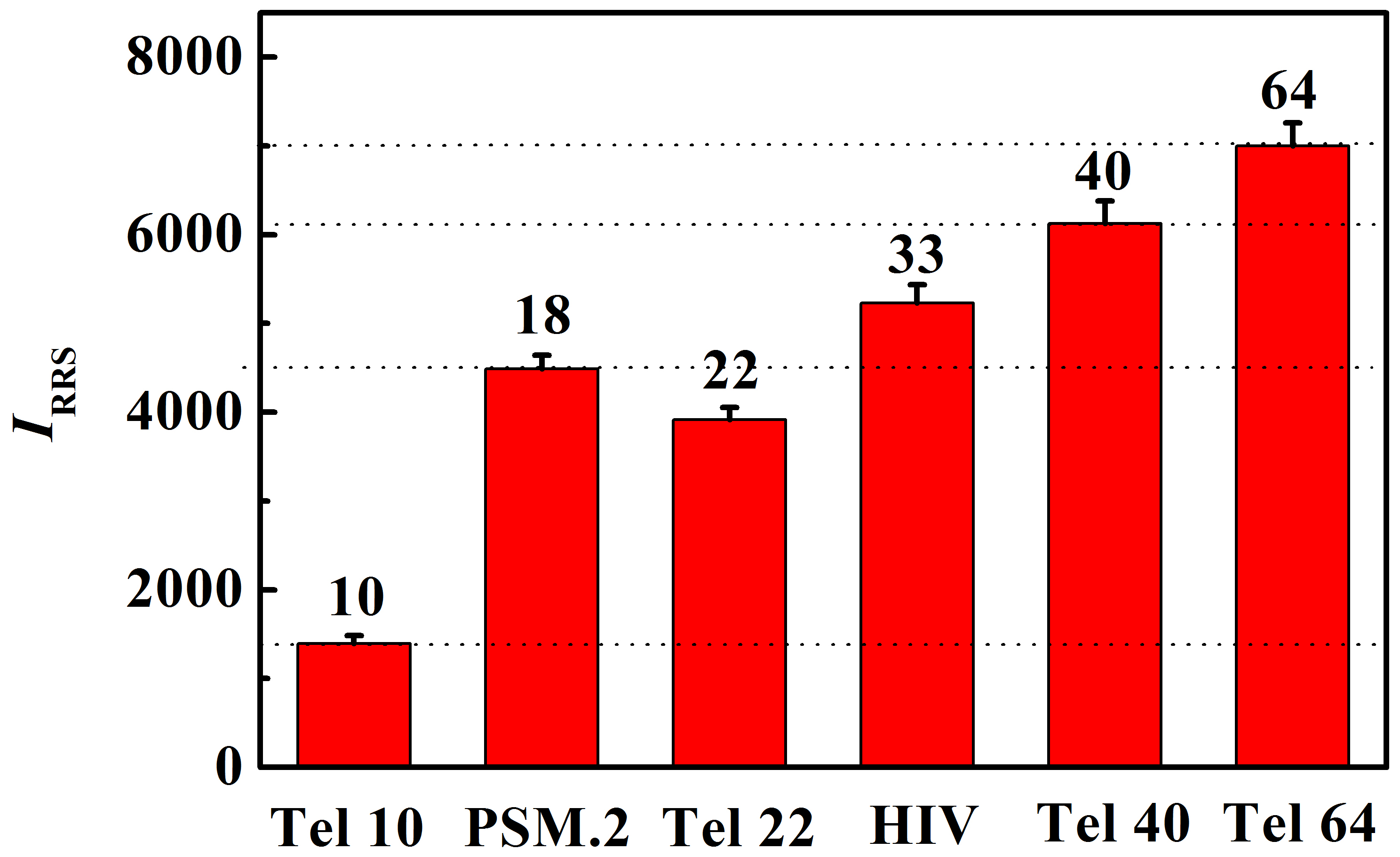
**

**Figure S14** Comparison of RRS signals of different PEI-Ag NCs/DNA system (Tel 10, PSM.2, Tel 22, HIV, Tel 40 and Tel 64). All concentrations of DNA are 30 nM.


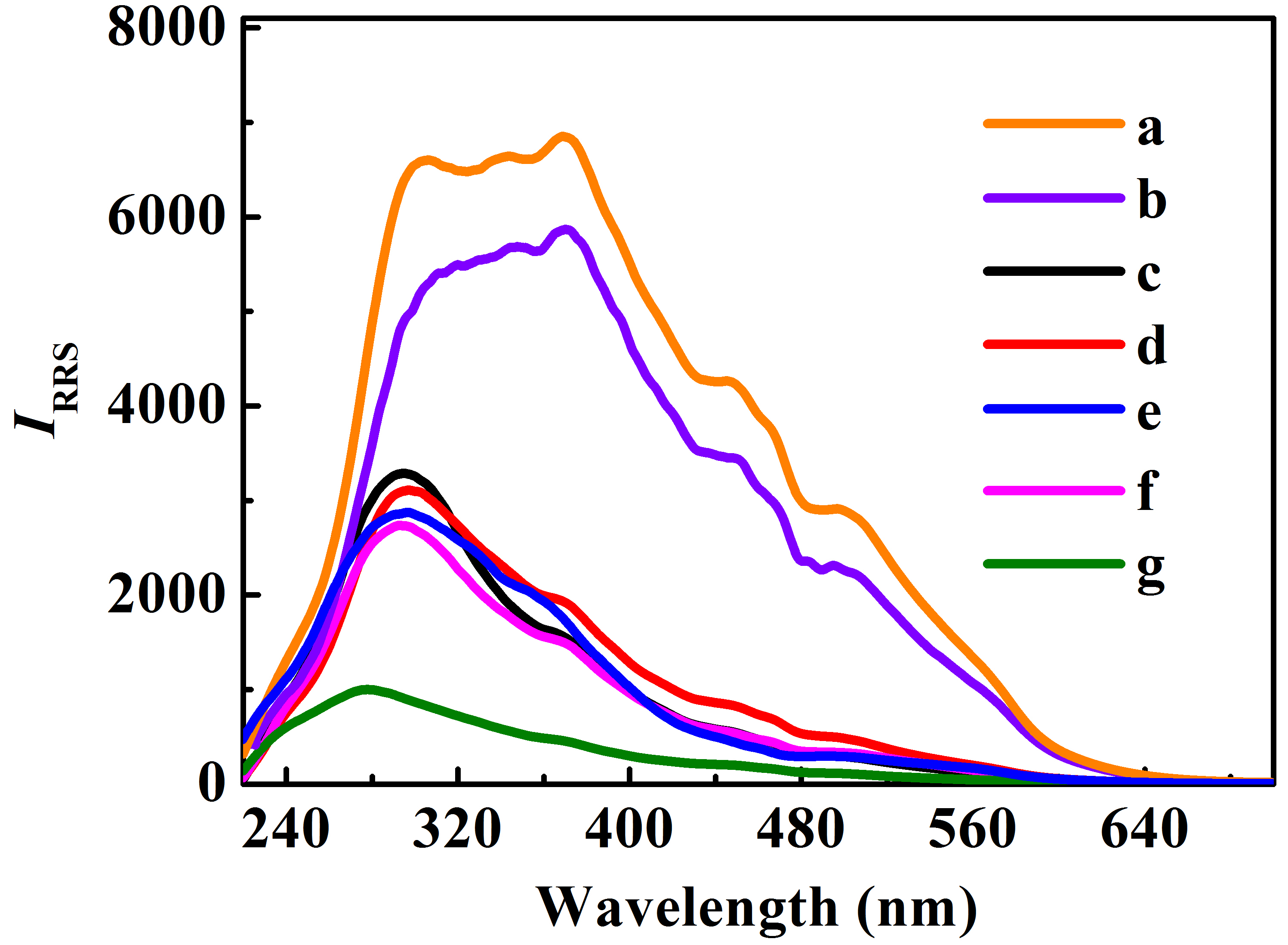


**Figure S15** Comparison of RRS spectra of PEI-Ag NCs, random coils, motifs and PEI-Ag NCs /motif system. (a) PEI-Ag NCs/C random coil (b) PEI-Ag NCs/G random coil (c) PEI-Ag NCs/G-quadruplex, (d) PEI-Ag NCs/i-motif, (e) G-quadruplex, (f) i-motif, (g) PEI-Ag NCs. The concentration of DNA in each case is 20 nM.

**
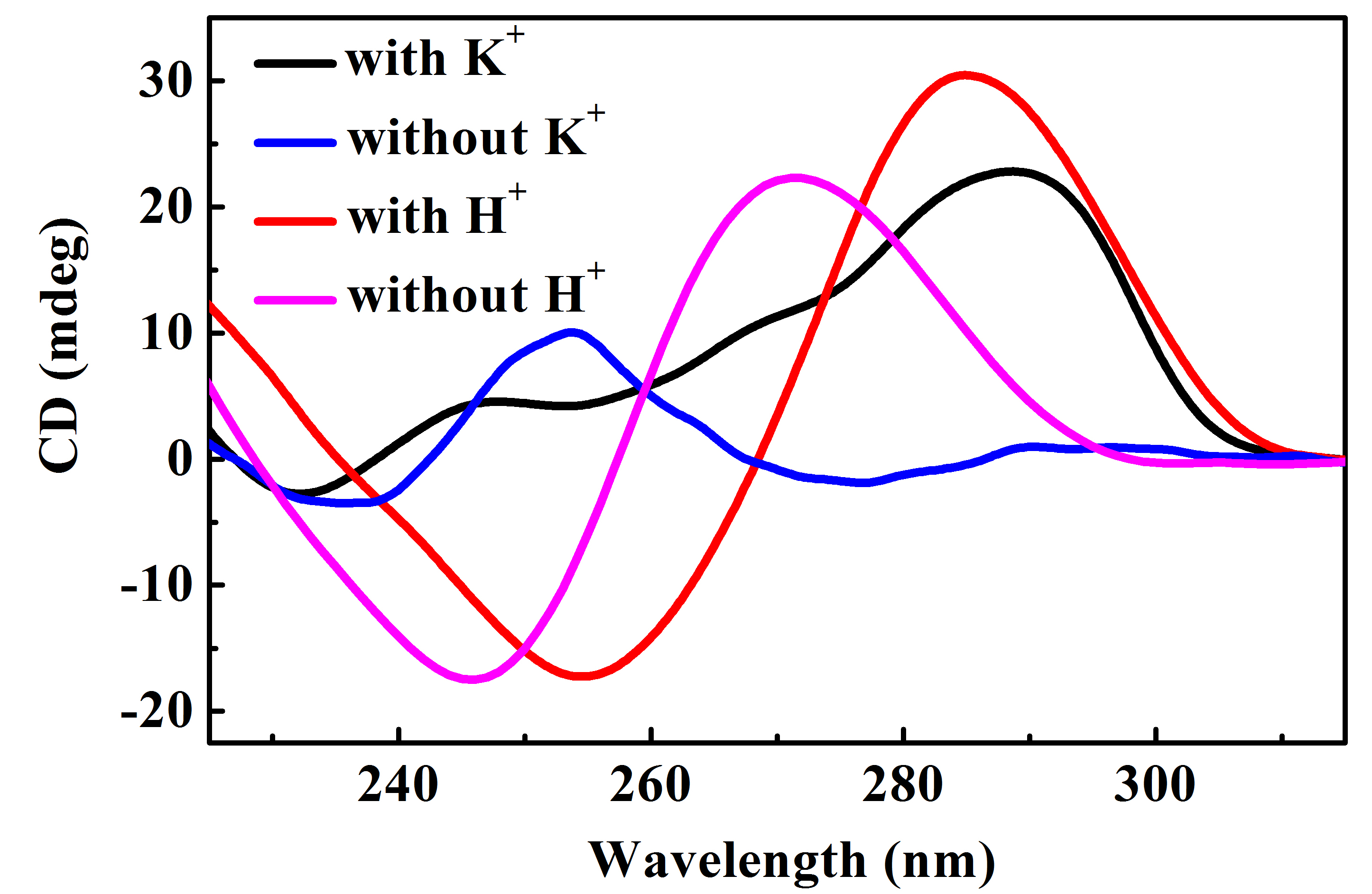
**

**Figure S16** CD spectra of G-rich strands and C-rich strands in the presence or absence of K^+^ or H^+^.

**
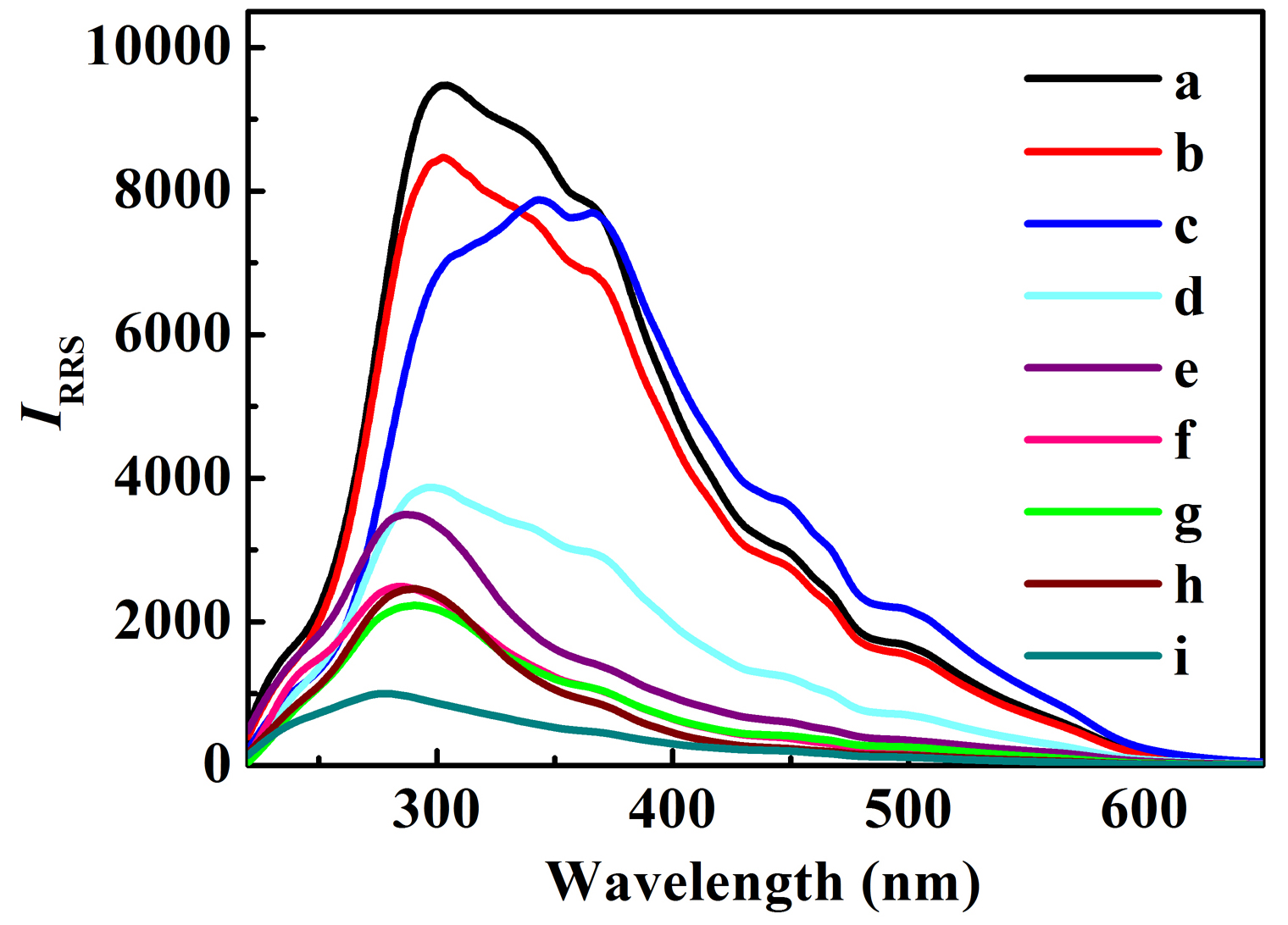
**

**Figure S17** RRS spectra of PEI-Ag NCs, DNA and PEI-Ag NCs/DNA system. (a) PEI-Ag NCs/duplex, (b) PEI-Ag NCs/i-motif/G random coil, (c) PEI-Ag NCs/ G-quadruplex/C random coil, (d) PEI-Ag NCs/G-quadruplex/i-motif, (e) duplex, (f) G-quadruplex and C random coil, (g) G-quadruplex/i-motif, (h) i-motif/G random coil, (i) PEI-Ag NCs. The total concentration of DNA in each case is 20 nM.

**
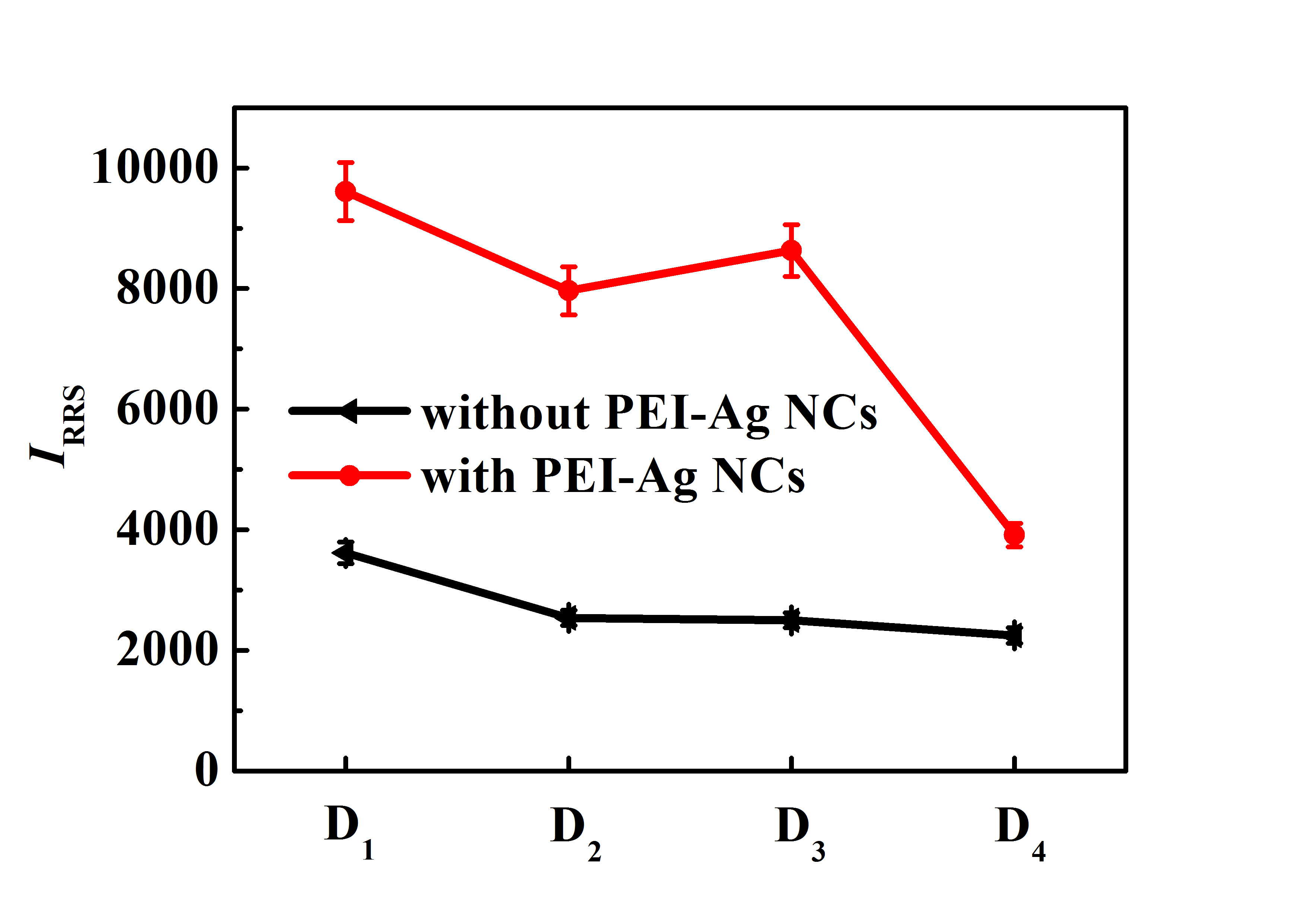
**

**Figure S18** RRS signals of DNA in absence and presence of PEI-Ag NCs. (D_1_) duplex, (D_2_) G-quadruplex and C random coil, (D_3_) i-motif and G random coil, (D_4_) G-quadruplex and i-motif. The total concentration of DNA in each case is 20 nM.

**Table S1** Oligonucleotides used in this work.

| Entry | Name | Sequence | The number of bases |
| --- | --- | --- | --- |
| 1 | Tel 10 | 5′-AG_3_T_2_AG_3_-3′ | 10 |
| 2 | Tel 22 | 5′-AG_3_(T_2_AG_3_)_3_-3′ | 22 |
| 3 | Tel 40 | 5′-AG_3_(T_2_AG_3_)_6_-3′ | 40 |
| 4 | Tel 64 | 5′-AG_3_(T_2_AG_3_)_10_-3′ | 64 |
| 5 | Ael 10 | 5′-C_3_TA_2_C_3_T-3′ | 10 |
| 6 | Ael 22 | 5′-C_3_T(A_2_C_3_T)_3_-3′ | 22 |
| 7 | Ael 40 | 5′-C_3_T(A_2_C_3_T)_6_-3′ | 40 |
| 8 | Ael 64 | 5′-C_3_T(A_2_C_3_T)_10_-3′ | 64 |
| 9 | PSM.2 | 5'-GTGGGTAGGGCGGGTTGG-3' | 18 |
| 10 | HIV | 5′-GCTATACATTCTTACTATTTTATTTAATCCCAG-3′ | 33 |

**Table S2** Reproducibility of RRS method

| Samples of PEI-Ag NCs | Inter-day precision CV% |
| --- | --- |
| 1 | 2.0 % |
| 2 | 3.3 % |
| 3 | 3.9 % |

**Table S****3** Linear ranges and correlation coefficients of the calibration graphs, and the detection limits for telomere DNA

| System | Linear range  (nM) | Linear regression equation | Correlation coefficient (R^2^) | LOD（nM） |
| --- | --- | --- | --- | --- |
| PEI-Ag NCs/Tel 10 | 20-400 | Δ*I*=12.12C+182.07 | 0.993 | 6.73 |
| PEI-Ag NCs/Tel 22 | 5-50 | Δ*I*=84.13C+322.58 | 0.994 | 0.97 |
| PEI-Ag NCs/Tel 40 | 0.7-20 | Δ*I*=194.10C+1082.30 | 0.990 | 0.42 |
|  | 20-70 | Δ*I*=72.45C+3137.50 | 0.991 |  |
| PEI-Ag NCs/Tel 64 | 0.3-1 | Δ*I*=661.40C+726.37 | 0.996 | 0.12 |
|  | 1-15 | Δ*I*=224.94C+1238.20 | 0.993 |  |
|  | 15-50 | Δ*I*=97.79C+3433.10 | 0.992 |  |
| PEI-Ag NCs/Ael 10 | 10-200 | Δ*I*=21.60C+477.21 | 0.998 | 3.77 |
| PEI-Ag NCs/Ael 22 | 5-50 | Δ*I*=88.34C+1330.10 | 0.990 | 0.92 |
| PEI-Ag NCs/Ael 40 | 1-70 | Δ*I*=99.90C+1844.00 | 0.985 | 0.81 |
| PEI-Ag NCs/Ael 64 | 0.5-40 | Δ*I*=229.10C+1028.8 | 0.991 | 0.36 |
